# HMGB1 as an extracellular pro-inflammatory cytokine: Implications for drug-induced organic damage

**DOI:** 10.1007/s10565-024-09893-2

**Published:** 2024-07-15

**Authors:** JianYe Yuan, Lin Guo, JiaTing Ma, HeJian Zhang, MingXuan Xiao, Ning Li, Hui Gong, Miao Yan

**Affiliations:** 1 Department of Pharmacy, the Second Xiangya Hospital, Central South University, Changsha, China; 2 Institute of Clinical Pharmacy, Central South University, Changsha, China; 3 International Research Center for Precision Medicine, Transformative Technology and Software Services, Hunan, China; 4 Xiangya School of Medicine, Central South University, Changsha, China; 5 Department of Pathology, The Eight Affiliated Hospital, Sun Yat-Sen University, Shenzhen, China; 6 Xiangya School of Pharmaceutical Sciences, Central South University, Changsha, China

**Keywords:** HMGB1, Drugs, Toxicity, Inflammation, Therapy

## Abstract

Drug-induced organic damage encompasses various intricate mechanisms, wherein HMGB1, a non-histone chromosome-binding protein, assumes a significant role as a pivotal hub gene. The regulatory functions of HMGB1 within the nucleus and extracellular milieu are interlinked. HMGB1 exerts a crucial regulatory influence on key biological processes including cell survival, inflammatory regulation, and immune response. HMGB1 can be released extracellularly from the cell during these processes, where it functions as a pro-inflammation cytokine. HMGB1 interacts with multiple cell membrane receptors, primarily Toll-like receptors (TLRs) and receptor for advanced glycation end products (RAGE), to stimulate immune cells and trigger inflammatory response. The excessive or uncontrolled HMGB1 release leads to heightened inflammatory responses and cellular demise, instigating inflammatory damage or exacerbating inflammation and cellular demise in different diseases. Therefore, a thorough review on the significance of HMGB1 in drug-induced organic damage is highly important for the advancement of pharmaceuticals, ensuring their effectiveness and safety in treating inflammation as well as immune-related diseases. In this review, we initially outline the characteristics and functions of HMGB1, emphasizing their relevance in disease pathology. Then, we comprehensively summarize the prospect of HMGB1 as a promising therapeutic target for treating drug-induced toxicity. Lastly, we discuss major challenges and propose potential avenues for advancing the development of HMGB1-based therapeutics.

**Graphical Abstract:**

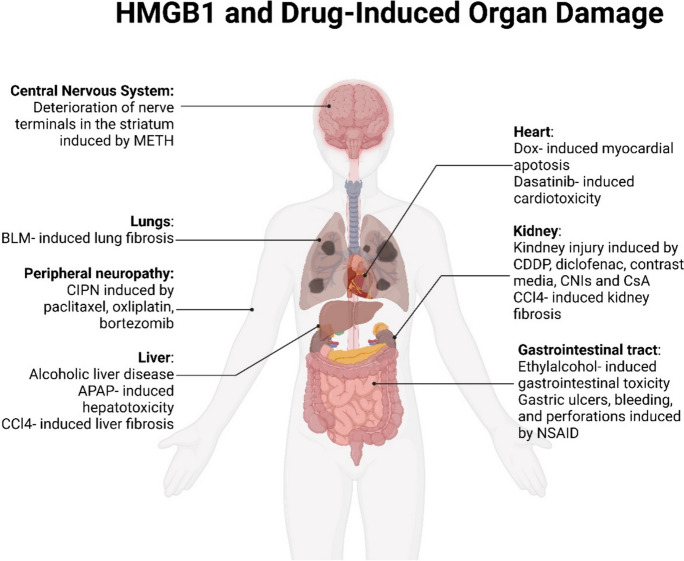

Graphical Headlights

(1) A comprehensive overview of the intricate relationship between HMGB1 and drug-induced organ toxicity is presented, accompanied by the corresponding treatment strategies.

(2) The present study addresses significant obstacles and suggests potential strategies for furthering the progress of HMGB1-based therapeutics.

(3) The research prospects of HMGB1 are also summarized.

## Introduction

Pharmaceutical substances possess the capacity to modulate physiological processes and serve as valuable tools in disease prevention, treatment, and diagnosis. However, it is important to acknowledge that drugs can also elicit toxic reactions, manifesting as physiological aberrations or even pathological alterations in organic structure. The utilization of numerous commonly employed medications is constrained by the potential for drug-induced toxicity to various organs. Recent research has demonstrated that high-mobility group box-1 (HMGB1) is an essential factor in many drug-induced organic injuries, thereby suggesting novel strategies to mitigate such adverse effects. HMGB1, a non-histone protein firstly identified during 1970s, is situated within the nucleus and can also be secreted. HMGB1 is involved in maintaining the integrity of nucleosomes and regulating various intranuclear biological processes such as DNA replication, transcription, recombination, and repair. Active secretion of HMGB1 mainly occurs in monocytes, natural killer cells, dendritic cells (DC), platelets, and endothelial cells. HMGB1 can also be passively released from the nucleus of necrotic cells. Extracellular HMGB1, by its interaction with several receptors, can induce damage to multiple organs, including the liver, lungs, gastrointestinal tract, and heart. In this study, we commence by providing an overview of the properties, functions, translocation, and secretion of HMGB1 in various diseases. Subsequently we summary the current research on the potential involvement of HMGB1 in the progression of drug-induced organic damage.

## HMGB1 overview

### Structure and regulation of HMGB1

HMGB1 is a non-histone protein that primarily existing within nucleus but may also be secreted (Andersson et al. 2022; Chen et al. 2004b; Dumitriu et al. 2009; Gardella et al. 2002). HMGB1 consists of 215 amino acids and contains a sour tail in C-terminus and two positively charged domains that bind to DNA (Box A and Box B) in N-terminus (Andersson and Tracey 2011; Harris et al. 2012). Box B (aa 95–163) has pro-inflammatory activity, containing binding sites for TLR4 and RAGE (Yang et al. 2015a). HMGB1 residues 89–108 interact with TLR4 and 150–183 with RAGE (Diener et al. 2013; Huttunen et al. 2002). Box A (aa 9–79) functions as a region that triggers an anti-inflammatory response. It contains two binding sites for heparin and proteolytic cleavage, serving as a natural antagonist of HMGB1 (Ellerman et al. 2007). The acidic C-terminal region (aa 179–185), is made up of 30 aspartate and glutamate residues. The DDDDE sequence in the acidic C-terminal region of HMGB1 can bind to core histone H3 and mediate the binding of HMGB1 to chromosomes and stimulate transcription (Ueda et al. 2004).

The immunological activity of HMGB1 is dictated by its hemivesic acid residues: cys23, cys45, and cys106. Forms of HMGB1, including the fully reduced form, partially oxidized form (disulfide form), and fully oxidized form (sulfonyl form), are determined by these residues (Tang et al. 2016). In its fully reduced form, HMGB1 has all 3 reduced forms of hemivesic acid residues, each with a sulfur side group (-SH). The disulfide form contains a disulfide bond linking cys23 and cys45 (R-S–S-R) and reduced form of cys106. The sulfonyl form of HMGB1 contains sulfonated cys23, cys45, and cys106 (Satoh 2022; Tang et al. 2016). Fully reduced form exists inside the nucleus (Satoh 2022) without capability to combine TLR4/Myeloid differentiation factor-2 (TLR4/MD-2) or induce the generation of cytokines. However, fully reduced form can combine with CXCL12 to form complexes These complexes can bind to CXCR4, resulting in chemokine secretion. Subsequently, this stimulates a mobilization of immune cells and other cells that promote tissue regeneration (Satoh 2022). HMGB1 in its oxidized form can bind to TLR4, triggering generation of inflammatory factors (Yang et al. 2010). The fully oxidized form has no ability to activate chemokines or cytokines or induce their secretion (Satoh 2022; Tang et al. 2016).

Phosphorylation, acetylation, and methylation can modulate the interaction between HMGB1 and DNA. Poly (ADP-Ribosyl) modification of HMGB1 inhibits macrophage efferocytosis (Davis et al. 2012). Acetylated HMGB1 promotes cytoplasmic translocation within neutral granulocytes (Ito et al. 2007). Acetylation of lysine residues in the nuclear localization signal (NLS) of activated monocytes induce the translocation of HMGB1 from the nucleus to the cytoplasm (Bonaldi et al. 2003; Lu et al. 2014a). Phosphorylated NLS induced by classical protein kinase C is the cytoplasmic localization signal for HMGB1 (Oh et al. 2009). Since HMGB1 lacks a leader sequence, it can only be released extracellularly through the non-classical lysosomal pathway (Gardella et al. 2002).

**Fig. 1:**
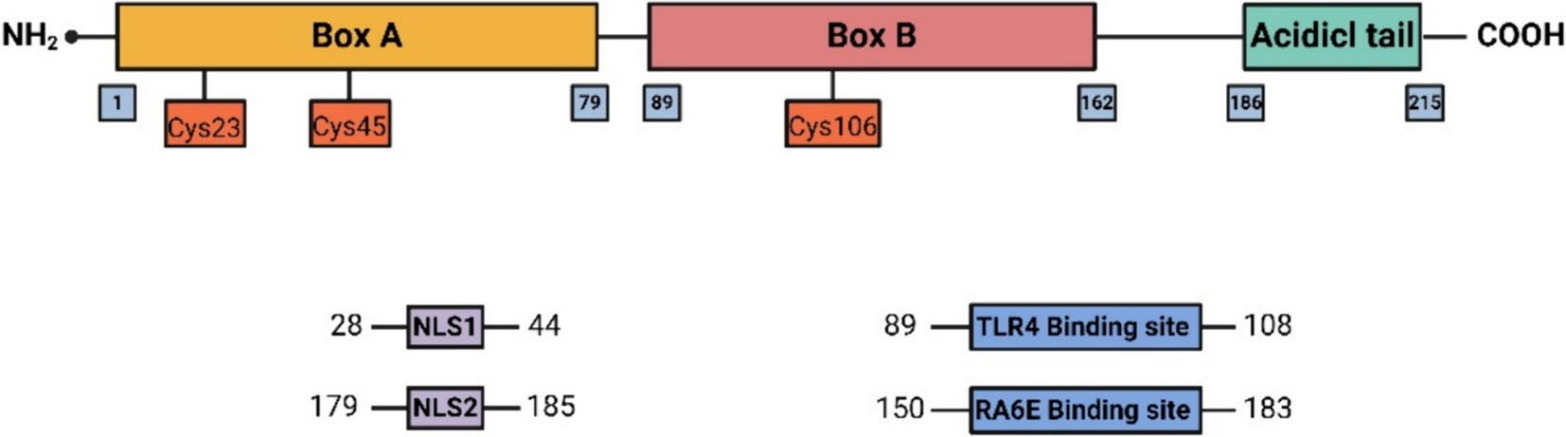
Structure and regulation of HMGB1. HMGB1 consists of 215 amino acids, consisting of Box A, Box B and acidic tail from N-terminus to C-terminus. Box A acts as an autoantagonist for HMGB1, while Box B contains binding sites for RAGE and TLR4. The acidic tail binds to chromosomes and stimulates transcription. The modification status of cys23, cys45 and cys106 determines the redox status and activity of HMGB1. The modification status of NLS determines the localization of HMGB1.This figure was drawn with Biorender (www.biorender.com)

In summary, the structure, redox status and modulation of HMGB1 all affects the secretion and function of HMGB1 (Fig. 1, Structure and regulation of HMGB1).

## Functions of HMGB1

### Functions of HMGB1 in nucleus and cytoplasm

Within the nucleus, HMGB1 can bind to and bend double-stranded DNA. Consequently, HMGB1 can enhance the binding affinity of transcription factors such as p53, Rb, NF-κB, etc. HMGB1 can selectively bind to damaged or distorted DNA sequences, facilitating the process of DNA repair. HMGB1 is involved in DNA replication, DNA repair and chromatin remodeling, etc. (Xue et al. 2021).

The main cytoplasmic functions of HMGB1 include regulating apoptosis, autophagy and mitochondrial metabolism (Tao et al. 2022). HMGB1 regulates the accumulation of cleaved caspase-3 (Narumi et al. 2015) and binds to Beclin1, which is involved in autophagy, thereby maintaining autophagy while limiting apoptosis (Tang et al. 2010). HMGB1 binds to Beclin1 and ATG5, suppressing calpain-induced cleavage, hence modulating the shift from autophagy to apoptosis in epithelial cells during inflammation (Zhu et al. 2015). Additionally, HMGB1 may inhibit the signaling mediated by STAT3, thus promoting autophagy and providing protection against infection in intestinal epithelial cells (Zhang et al. 2019). HMGB1 enhances the inhibitory role of p53 in non-alcoholic fatty liver disease (NAFLD)-induced autophagy and thus promotes NAFLD-induced autophagy. The HMGB1 nucleus to cytoplasm relocation and the subsequently upregulated Beclin1 expression is essential in the NAFLD (Zhang et al. 2020).

### Extracellular functions of HMGB1 and receptors

**Fig. 2:**
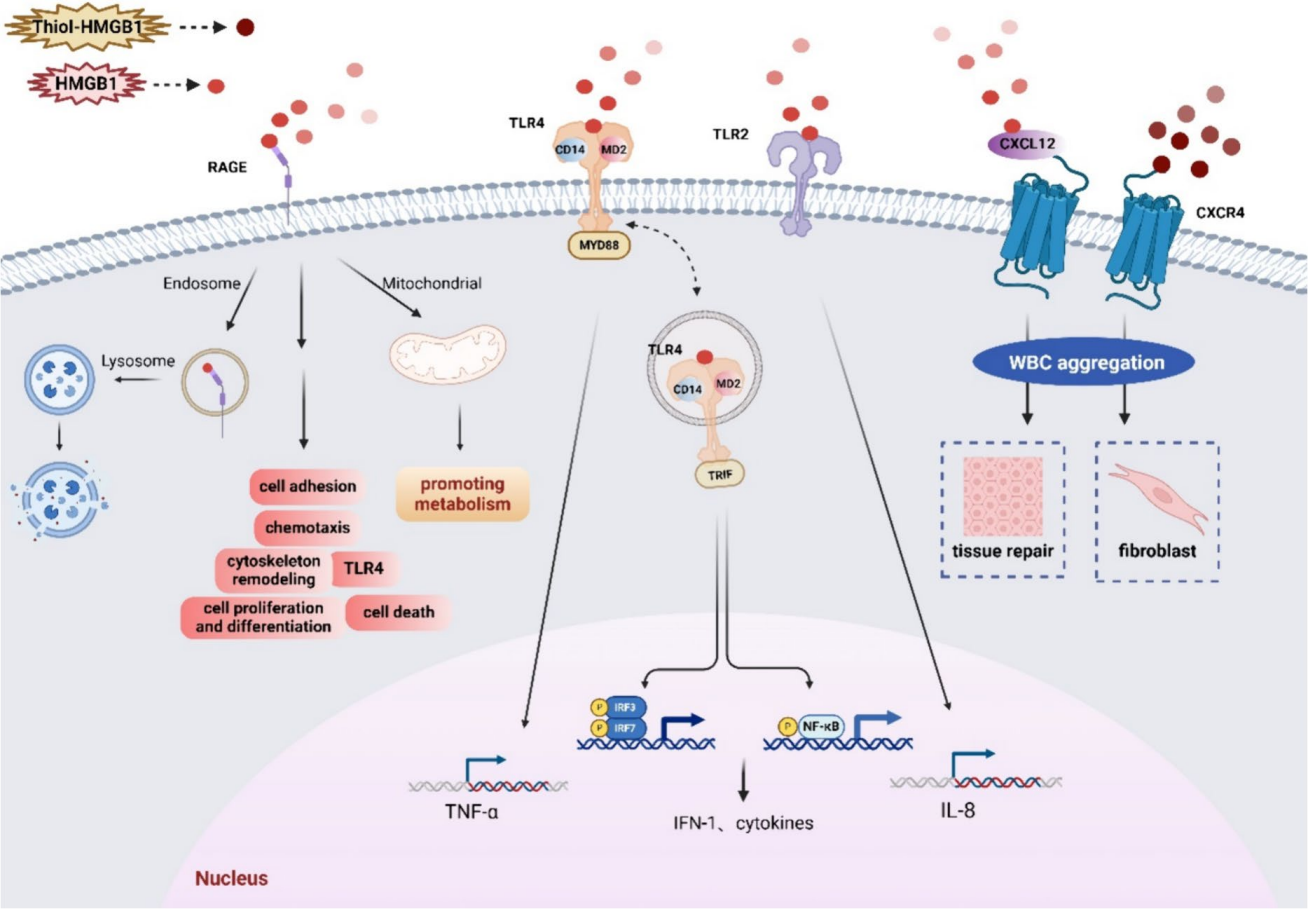
Extracellular Functions of HMGB1 and Receptors. The most important receptors of HMGB1 include RAGE, TLR4 and CXCR4. 1) The interaction between HMGB1 and RAGE can affect the cell's lysosomal system and mitochondria, thereby affecting cytoskeletal remodeling, cell adhesion, cell chemotaxis, apoptosis, cell proliferation and differentiation. 2) The interaction between HMGB1 and TLR4 can trigger MyD88 to increase the expression levels of inflammatory factors such as IFN and TNF-α by stimulating NF-κB and IRF. MD-2 and CD14 are critical for the binding of HMGB1 and TLR4. HMGB1 can also stimulate TLR2 to increase the expression level of IL-8. 3) Under normal circumstances, HMGB1 must first bind to CXCL12 before interacting with CXCR4. The interaction between HMGB1 and CXCR4 can stimulate leukocyte migration, thereby further promoting tissue repair and fibrosis. This figure was drawn with Biorender (www.biorender.com)

HMGB1, which can be considered a damage-associated molecular pattern molecule (DAMP), functions as a pro-inflammatory factor. HMGB1 binds to several receptors, including RAGE, TLRs (TLR2, TLR4, TLR9), integrin, CD24, CXCR4, and N-methyl-d-aspartate receptor (NMDAR) (Kang et al. 2014a), which may be affected by co-factors. The CXCL12 complex (also called stromal cell-derived factor 1, SDF-1) is necessary for the interaction between HMGB1 and CXCR4 (Fig. 2, Extracellular Functions of HMGB1 and Receptors) (Schiraldi et al. 2012). HMGB1, CD24 as well as Siglec-10 form trimeric complexes, which can inhibit inflammation induced by acetaminophen (AAP) via the NF-κB signaling pathway (Chen et al. 2009). The DNA-containing immunological complex, formed by the combination of HMGB1 and CpG oligodeoxynucleotides, enhances cytokine production by activating RAGE and TLR-9 receptors (Tian et al. 2007). Below, we will provide detailed information on three main receptors of HMGB1.

RAGE, the firstly discovered HMGB1 receptor, belongs to the immunoglobulin superfamily (Hori et al. 1995). RAGE consists of a transmembrane domain, an extracellular domain, and a cytoplasmic tail (Schmidt et al. 2001). Extracellular region of RAGE includes 3 immunoglobulin-like domains (Schmidt et al. 2001). The distal V-type domain is accountable for ligand binding, while the cytosolic tail is crucial for intracellular signaling mediated by RAGE (Schmidt et al. 2001). RAGE functions in several biological processes with other HMGB1 receptors, including cytoskeletal remodeling, cells adhesion, chemotaxis, apoptosis, proliferation and differentiation (Xue et al. 2021). The HMGB1-RAGE pathway is vital in chronic disorders associated with oxidative stress, as well as in promoting tumor growth and invasion (Ibrahim et al. 2013; Taguchi et al. 2000). HMGB1 can enter the lysosomal and endosomal system with its partner molecules via interacting with RAGE (Xu et al. 2014; Yang et al. 2016). HMGB1 can interfere with the integrity of the lysosomal membrane in acidic conditions (Xu et al. 2014). As a result, HMGB1 and its partner molecules enter the cytoplasm, leading to inflammasome activation, pyroptosis, and release of proinflammatory mediators (Andersson et al. 2018). RAGE is also found inside the mitochondria of cancer cells (Kang et al. 2014b). The HMGB1-RAGE interaction modulates cellular metabolism and promotes tumor proliferation by increasing the generation of ATP in the mitochondria (Xue et al. 2021).

TLRs are highly conserved proteins and have leucine-rich repeats (LRR) in the extracellular part and Toll/IL-1 receptor (TIR) within cytoplasm, which is capable of initiating the innate immune response (Taverna et al. 2022). Interaction between TLRs and HMGB1 triggers cytokines and chemokines secretion through interferon regulatory factor (IRF) and NF-κB (Xue et al. 2021). Specific binding of HMGB1 and TLR4 is highly relevant for TNF-induced inflammatory response (Venereau et al. 2012). Myeloid differentiation factor 2 (MD-2) is vital in the interaction between TLR4 and disulfide HMGB1, potentially explaining TLR4's high affinity to this specific form of HMGB1 (Satoh 2022; Yang et al. 2015b). TLR4 triggers myeloid differentiation primary response 88 (MyD88) as well as TIR-domain-containing adapter-inducing interferon-β (TRIF), causing inflammatory factors generation and type I interferon (IFN) via stimulating NF-κB and interferon regulatory factors (IRF)3 and IRF7 (Satoh 2022). The activation of TLR4/MD-2 complex necessitates CD14 in macrophages (Satoh 2022). HMGB1 selectively stimulates the release of IL-8 only from TLR2. When treated with TLR2 antagonists, HEK293/TLR2-expressing cells, which have been pre-treated with HMGB1, show a decrease in IL-8 production (Yu et al. 2006). HMGB1 enhances neutrophil extracellular trap (NETs) formation mainly through interaction with TLR4. In this process, the TLR4-MyD88 signaling pathway plays an important role. In addition, activation of NADPH oxidase and stimulation of TLR2 may play a secondary role. Neutrophil binding to HMGB1 increases the interaction between neutrophil elastase and myeloperoxidase. But the role of myeloperoxidase in DNA release is not yet clear. LPS-treated mice receiving HMGB1-neutralizing antibodies had reductions in histone 3 and cell-free DNA in the BAL fluid, which are markers of neutrophil extracellular traps (NTEs) attenuation (Tadie et al. 2013). Furthermore, during the course of NETs, DNA enhanced the proteolytic cleavage of HMGB1 by elastase, which enhances binding activities of the HMGB1 towards G-quadruplex DNA, holliday junction DNA, and TLR4•MD-2 complex (Wang et al. 2022). NETs also induce Kupffer cell M1 polarization and reduce intracellular translocation of HMGB1 by inhibiting DNase-1 (Liu et al. 2022). Neutralizing HMGB1 may help inhibit NETs and direct toxicity of NETs as well as cell death (Zhan et al. 2022).

CXCR4, a G protein-coupled receptor (GPCR), is extensively found in hematopoietic cells as well as tumor tissues (Kang et al. 2014a). Thiol-HMGB1 can combine with CXCL12 to form a heterocomplex. The complex then binds to CXCR4 (Schiraldi et al. 2012). This pathway is vital to the balance of lymphoid organs and promoting the migration of white blood cells to injured tissues, hence facilitating tissue healing without exacerbating inflammation (Tirone et al. 2018). A recent finding has revealed that HMGB1-3 s, a special form of HMGB1, in which non-oxidizable serine substitutes for all three cysteine residues, can interact with CXCR4 without CXCL12 and stimulate cardiac fibroblast migration in the context of myocardial infarction, worsening tissue remodeling after myocardial infarction (Di Maggio et al. 2017).

## HMGB1 translocation and secretion

### HMGB1 nucleus to cytoplasm translocation

HMGB1 possesses NLS1 at aa 28–44 and NLS2 at aa 179–185 as well as two non-classical nuclear export signals (NESs) (Bonaldi et al. 2003). Because of the two NLSs, HMGB1 typically locates within the nucleus (Bonaldi et al. 2003). However, HMGB1 moves between the nucleus and cytoplasm via altering NLSs (Xue et al. 2021). IFN-β can induce HMGB1 nucleus to cytoplasm translocation via activating the JAK/STAT1 pathway through hyper-acetylated NLSs (Lu et al. 2014a). Phosphorylation or methylation of NLSs may also enhance HMGB1 accumulation in cytoplasm (Tang et al. 2016). XPO1, belonging to importin β superfamily of nuclear transport receptors (Turner et al. 2012), can recognize and export proteins with NESs. XPO1 can facilitate the extranuclear transfer of RNA or proteins (Turner et al. 2012; Turner and Sullivan 2018) including HMGB1 (Kwak et al. 2019).

### Active HMGB1 extracellular secretion

Active HMGB1 extracellular secretion does not occur through the traditional endoplasmic reticulum-Golgi secretory route because of lacking a leader sequence (Kwak et al. 2020; Palade 1975). Triggered by microbial products like LPS, infections or endogenous host stimuli, active HMGB1 extracellular secretion occurs (Cai et al. 2019; Wang et al. 1999) via intracellular vesicles. HMGB1 can be packaged into lysosomes or autophagosomes. Afterwards, fusion of vesicles with the cytoplasmic membrane enables HMGB1 extracellular secretion (Lu et al. 2014b; Pisetsky 2014). Immune cells, endothelial cells, platelets, neurons, cancer cells, and astrocytes. are the primary source of HMGB1. Table 1 (Active HMGB1 Extracellular Secretion) presents a collection of commonly documented mechanisms for the active release of HMGB1.

**Table 1:** Active HMGB1 Extracellular Secretion

Stimulus	Inhibitors	Mechanism	Main Secretory Cell	REF
Oxidative stress (ROS)	NFE2-HMOX1	MAPK1 and NF-κB	Macrophages, Monocytes	(Mazur-Bialy and Pocheć 2021; Tang et al. 2007b)
NOC-18, RNS	CORM-2	IFNB-JAK2-STAT1	Macrophages	(Tsoyi et al. 2010)
Calcium ions	Calcium signaling inhibitors, Calcium chelators	Ca2 + / CaMKK, IFNB	-	(Ma et al. 2012; Shin et al. 2014; Tsoyi et al. 2010; Zhang et al. 2008; Zhao et al. 2017)
LPS, TNF, XPO1	HSP72	-	Macrophages	(Kwak et al. 2019; Tang et al. 2007a)
LPS, TNF	-	LPS-CD14-TNF,	Macrophages, Monocytes	(Chen et al. 2004a)
LPS, Notch	DAPT	JNK	Macrophages	(Tsao et al. 2011)
STAT1, STAT3	-	JAK-STAT	-	(Imbaby et al. 2020; Park et al. 2018)
TP53	-	-	Cancer Cells	(Luo et al. 2018a)
DAMPs, Inflammasomes	C5aR2	Caspase-1, Caspase-11, IL1, PKM2- NLRP3	Immune Cells, Cancer Cells	(Barlan et al. 2011; Craven et al. 2009; Lamkanfi et al. 2010; Miller et al. 2014)
Secretory Lysosomes, IFI30	-	-	Immune Cells, Fibroblasts	(Chiang and Maric 2011)
Alkaliptosis	-	-	-	(Fang et al. 2021)

### Passive release of HMGB1

Passive Release of HMGB1 occurs after processes including necrosis, apoptosis, NETosis, pyroptosis, ferroptosis, autophagy-dependent cell death, etc., reacting to diverse stressors or damage (Chen et al. 2022). Table 2 (Passive Release of HMGB1) presents a collection of commonly documented mechanisms for the passive release of HMGB1.

**Table 2:** Passive Release of HMGB

Stimulus	Inhibitors	Mechanism	Main Secretory Cell	REF
Extensive DNA Damage, PARP1	-	-	-	(Ni et al. 2020; Qin et al. 2015)
Cytokines, Infection, Chemotherapy, RIPK3	Necrostatin-1	Necroptosis	-	(Liu et al. 2021b; Murakami et al. 2014)
Pathogen Invasion, Chemotherapy, Cathepsin	-	Necrosis, Apoptosis, Ferroptosis	-	(Tang et al. 2019)
Oxidative Stress, ROS	-	Necrosis, Apoptosis, Necroptosis, Ferroptosis	-	(Chen et al. 2021)
Apoptotic Cells, DNase	DR396	NETosis	Neutrophils	(Tohme et al. 2016)
Caspase, Cytochrome c, p57,	-	Apoptosis, Pyroptosis	-	(Chen et al. 2022)
Autophagy-Related Proteins	-	Autophagy-Dependent Cell Death	-	(New and Thomas 2019; Wen et al. 2019)

### HMGB1 and drug- induced organic injury

Drug- induced toxicity mediated by HMGB1 occurs in multiple organs, especially in the liver and kidney due to their crucial roles in the pharmacokinetics and elimination of drugs. HMGB1- mediated drug- induced injury to the peripheral nervous system and myocardium is also common. The subsequent sections will provide detailed mechanisms of drug- induced toxicity related to HMGB1 in diverse organic injury and potential treatment strategies.

### HMGB1 and drug- induced liver injury (DILI)

**Fig. 3:**
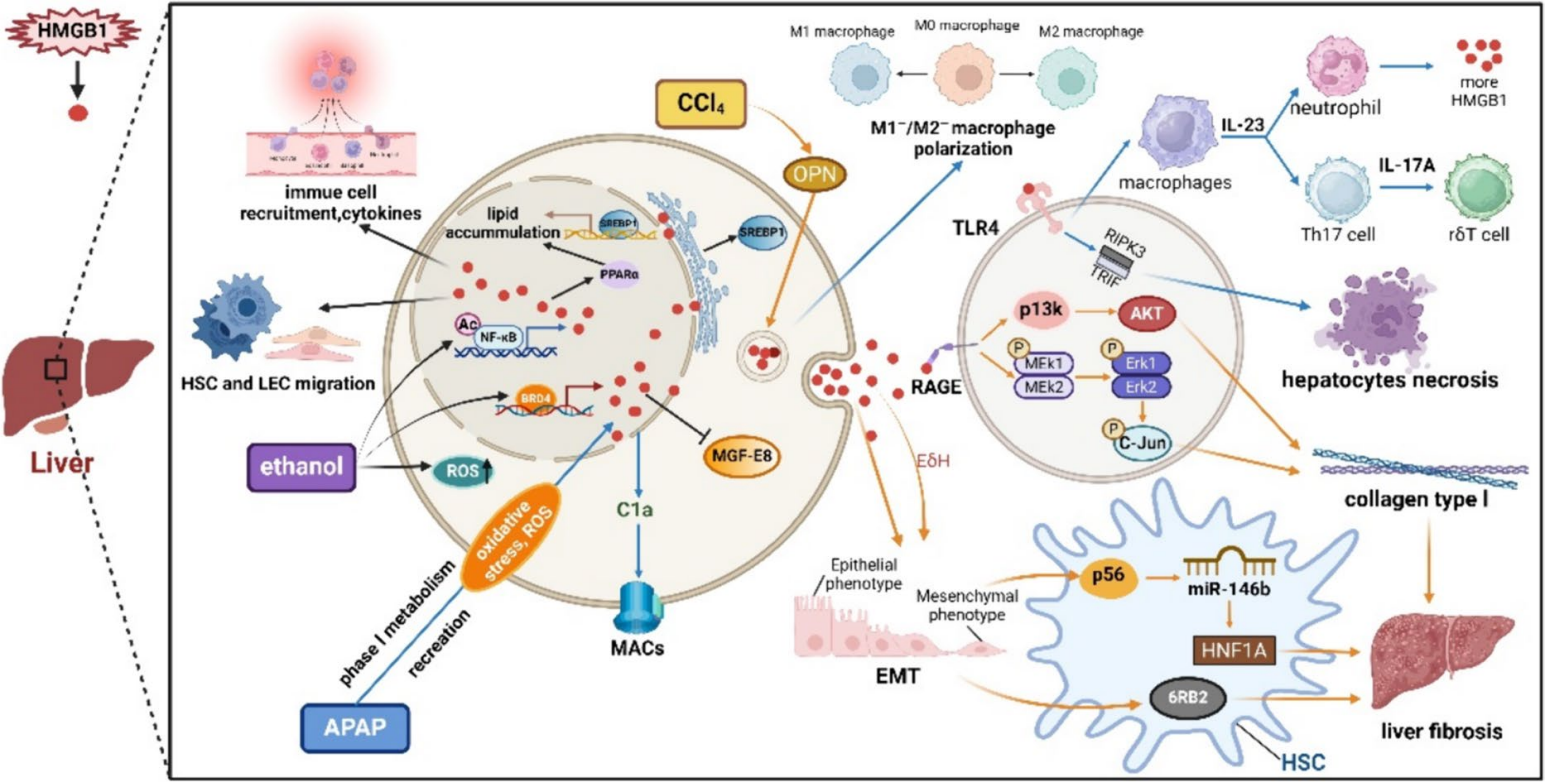
HMGB1 and DILI. HMGB1 is involved in liver injury induced by substances such as ethanol, APAP, and CCl4. 1) Ethanol upregulates HMGB1 levels by promoting NF-κB acetylation, ROS formation and increasing bromodomain-containing protein 4 (BRD4). The processes promoted by alcohol- induced HMGB1 include SREBP1 and PPAR-mediated fatty liver formation, hepatic stellate cells (HSC) and liver endothelial cells (LEC) migration, immune cell recruitment and release of inflammatory factors, and MFG- E8 inhibition. 2) APAP increases ROS levels through phase I metabolic reaction, thereby upregulating HMGB1 levels. The following phenomenon were observed in APAP- induced liver injury. HMGB1 promotes M1 macrophage- mediated liver injury. HMGB1 further stimulates the development of IL-17A-producing γδ T cells and neutrophil infiltration by promoting IL-23 production. HMGB1 also induces necrosis of adjacent hepatocytes by stimulating the TLR4-TRIF-RIPK3 pathway. Finally, HMGB1 binds to C1q and induces the formation of MACs. 3) HMGB1 is involved in the process of CCl4- induced liver fibrosis, which is promoted by (Osteopontin) OPN. The following phenomenon occur in CCl4- induced liver fibrosis. HMGB1 promotes epithelial-mesenchymal transition (EMT). HMGB1 also activates the pMEK1/2/pERK1/2/pcJun and PI3K/Akt axis by interacting with RAGE to promote the production of Collagen type I. HMGB1 upregulates the expression of miR-146b in a p65- dependent manner to target HNF1A and downregulate its expression. These processes all promote liver fibrosis. This figure was drawn with Biorender (www.biorender.com)

Liver injury is a commonly observed side effect in clinical trials. Severe liver injury has been a direct factor leading to the suspension of new drug research and the decline in pharmaceutical sales (Kaplowitz 2005). Damaged hepatocytes have the potential to initiate inflammation through the release of various mediators. DAMPs, especially HMGB1, plays a vital role in DILI (Fig. 3, HMGB1 and DILI).

### HMGB1 and ethylalcohol- induced hepatotoxicity (alcoholic liver disease, ALD)

ALD serves as a common origin for chronic hepatic damage. The pathophysiology of ALD encompasses alcoholic steatosis, hepatitis, liver fibrosis, cirrhosis, etc. (Gustot and Jalan 2019). Liver biopsies in ALD reveal upregulated HMGB1 expression and stimulation of HMGB1 translocation, indicating a positive association with disease severity compared to healthy liver explants (Ge et al. 2014). Hepatocytes from ethanol‐ fed mice have shown an improved ability to stimulate nucleus to cytoplasm translocation and secrete HMGB1, surpassing the capacity of Kupffer cells (Gaskell et al. 2018). Ethanol hinders NF-κB translation and but promotes NF-κB acetylation, resulting in increased production of enzymes generating reactive oxygen species (ROS). These enzymes act as triggers for the extracellular secretion of HMGB1, subsequently contributing to liver inflammation (Zhou et al. 2020) and fibrosis (Lee et al. 2019). Alcohol exposure augments lipid accumulation via regulating SREBP1, a transcription factor, and PPARα, a nuclear hormone receptor. PPARα and SREBP1 govern lipid synthesis and β-oxidation in the liver (Cioarca-Nedelcu et al. 2021). Targeted elimination of HMGB1 provided protection against ALD in mouse hepatocytes (Khambu et al. 2019). HMGB1 knockdown reduces SREBP-1 synthesis and lipids accumulation (Gaskell et al. 2018), underscoring the significance of HMGB1 secretion in hepatocytes in ALD development. Furthermore, extracellular HMGB1 prompts the migration of HSC and LEC (Seo et al. 2013), orchestrating immune cell recruitment and subsequent release of inflammatory factors in ALD (Khambu et al. 2019). In ALD, milk fat globule-EGF factor 8 protein (MFG-E8), a vital molecule in macrophage-mediated phagocytosis of apoptotic cells, shows a strong interaction selectivity for αvβ3 integrin on macrophages and Phosphatidylserine (PtdSer) on apoptotic cells membrances. MFG-E8 may enhance macrophage efferocytosis through bridging macrophages to apoptotic cells. In contrast, HMGB1 can impede the binding of MFG-E8 to these structures (Wang et al. 2013a). Directly targeting HMGB1, BRD4, a member of Bromo and Extra-Terminal (BET) domain family, is significantly upregulated in ALD. HMGB1 acts a downstream inflammatory mediator of BRD4 in alcohol-induced liver injury. The BRD4/HMGB1 pathway may be implicated in ALD pathogenesis (Lan et al. 2020).

Digitoflavone (DG), a natural flavonoid found in various plants, can decrease macrophages and neutrophils infiltration. DG increases the suppressive effect of TLR4 or HMGB1 knockdown during inflammation induced by LPS/ATP. Betaine and SIRT1 overexpression also have similar effect (Zhao et al. 2022). DG can reverse liver inflammation by suppressing the HMGB1-TLR4 signaling pathway, interfering with NLRP3 inflammasome assembly, and reducing pro-inflammatory cytokines secreted by macrophages. Additionally, DG reverses the accumulation of lipids resulting from chronic alcohol exposure via inhibiting SREBP1 and PPARα, which are stimulators of extracellular secretion of HMGB1. Ultimately, DG shows promising therapeutic potential for treating ALD (Shang et al. 2022). Salvianic acid A (SAA), a phenolic acid identified from herbs, has been shown to significantly reduce the increase of BRD4 induced by alcohol and increasing inflammatory genes expression dose-dependently both in vivo and in vitro (Lan et al. 2020). Further research is needed to determine if SAA decreases the production of BDR4 via inhibiting transcription or by co-regulating the stability of BDR4 protein with alcohol.

### HMGB1 and acetaminophen (APAP)-induced hepatotoxicity

APAP, also known as paracetamol, is a well-recognized and extensively used NSAIDs. APAP has been shown to potentially cause DILI (Pu et al. 2019). In adults, phase II metabolic reactions (glucuronidation, sulfation) are the main metabolic degradation pathway of APAP (Davis et al. 1976; Steventon et al. 1996). Phase I metabolic reactions are the secondary metabolic degradation pathway (Zaher et al. 1998), inducing the generation of oxidative stress as well as ROS synthesis in hepatocytes mitochondria (Hu et al. 2016; McGill et al. 2012). Among the cytochrome P450 enzymes, CYP2E1 is the major enzyme involved in the phase I metabolism of APAP. APAP is converted through phase I reactions into NAPQI, which can covalently bind to proteins, leading to liver damage. At the same time, the metabolism of APAP through phase I reactions will consume a large amount of Glutathione (GSH), ultimately leading to generation of ROS synthesis in hepatocytes mitochondria, triggering the subsequent signaling events characteristic of APAP-induced programmed necrosis (Jaeschke et al. 2021). Necrotic hepatocytes secret multiple kinds of DAMPs, including HMGB1 (Antoine et al. 2009; Martin-Murphy et al. 2010). The release of HMGB1 might be conditional upon the stimulation of Caspase-1 (Liu et al. 2021a). M1 and M2 macrophages polarization is observed in APAP-mediated liver injury. M1 macrophages play a role in the early stage of APAP induction, producing pro-inflammatory factors such as IFN-γ, TNF-α, IL-1β, and IL-6, leading to inflammatory damage in the liver. M2 macrophages act mainly in the late stage, producing anti-inflammatory factors such as IL-4, IL-10 and TGF-β to promote tissue remodeling and fibrosis. Both kinds of macrophages express RAGE. HMGB1 is involved in M1-mediated APAP-induced liver injury. HMGB1 also epigenetically promotes the expression of IL-10 in M2 macrophages through RAGE (Rojas et al. 2018; Rojas et al. 2016; Tsuji et al. 2020). HMGB1 promotes the synthesis of IL-23 in hepatic macrophages via TLR4. Additionally, in the liver, IL-23 generated from macrophages contributes to the development of IL-17A-generating γδ T cells, which promote neutrophil infiltration after administrating APAP. However, the HMGB1 suppressant glycyrrhizin significantly reduces the synthesis of IL-17A and IL-23, as well as hepatic neutrophil accumulation (Wang et al. 2013b). As a consequence, there is an increased generation of neutrophils, leading to HMGB1 secretion. Eliminating neutrophils or suppressing the neutrophil extracellular traps (NETs) deregulates HMGB1 and inhibits hepatocellular necrosis (Liu et al. 2021a). APAP-induced injury mediated by HMBG1 induces necrosis of adjacent hepatocytes through the TLR4-TRIF-RIPK3-pathway (Minsart et al. 2020). Additionally, HMGB1 may bind to C1q and activate the classical complement pathway, ultimately inducing membrane attack complex (MACs) (Kim et al. 2018). A HMGB1 neutralizing antibody, h2G7, has been proved to reduce liver cell death and inflammation caused by excess APAP (Lundbäck et al. 2016). Runkuan Yang et al. discovered that neutralizing HMGB1 led to an upregulation of cyclin D1 expression in hepatocytes, indicating a potential enhancement of hepatocyte regeneration (Yang et al. 2012). This may be associated with the beneficial response induced via stimulating the NF-κB signaling pathway.

Metformin has been observed to bind to endogenous HMGB1 via the acidic tail, inhibiting its function in APAP-induced liver damage. In addition, metformin has the potential to influence the chromatin regulatory activity of intracellular HMGB1, specifically at the site where metformin is absorbed (Horiuchi et al. 2017). Glycyrrhetinic acid (GA) has been presented to have hepatoprotective effects. The primary mechanism through which GA achieves its hepatoprotective effect may involve inhibiting the release of HMGB1 and the consequent activation of TLR4-IRAK1-MAPK/NFκB axis. Additionally, it is possible to partially achieve this by inhibiting the expression of CYP2E1, which subsequently increases the hepatic GSH level and decreases ROS generation (Yang et al. 2017). Changchun Cai et al. demonstrated that Benzyl alcohol (BA) alleviated the APAP-induced liver injury. BA can decrease the production of inflammatory molecules, including keratinocyte-derived chemokine, IL-6, HMGB1 and IP-10, in mice serum. This protective effect of BA may be mediated by suppressing TLR4. BA also protects mitochondria in hepatocytes mainly by limiting APAP-induced JNK phosphorylation. Diacerein (DIA), capsaicin (CAP) and Kaempferol (KA) from Penthorum Chinense Pursh may has similar effect. Furthermore, the protective impact of DIA is partially achieved by the enhanced generation of PPAR-γ, which is analogous to DAPT, a γ-secretase inhibitor that effectively hinders Notch signaling (Cai et al. 2014). Apoptosis repressor with caspase recruitment domain (ARC) inhibits apoptosis. Junfeng An et al. shown that ARC effectively decreased the release of HMGB1 (An et al. 2013). Furthermore, there are several other drugs that reduce or alleviate HMGB1-mediated APAP-induced liver injury via various pathways. Liuweiwuling tablets have been shown to reduce the generation of HMGB1, IL-1β, as well as TNF-α, thus facilitating hepatic recovery in mice (Lei et al. 2015). Berberine (BBR), an alkaloid derived from Rhizoma Coptidis, has the ability to prevent the increase of HMGB-1 and phosphorylated NF-κB induced by APAP, as well as decrease the generation of inflammatory cytokines (Zhao et al. 2018). Chikusetsusaponin V (CKV) has the ability to regulate the generation of neutrophil NETs, as well as prevent the secretion of the endogenous HMGB1 and Caspase-1 stimulation (Liu et al. 2021a). HS octadecasaccharide (18-mer-HP or hepatoprotective 18-mer) provides protection against APAP-induced ALF through deregulating HMGB1/RAGE pathway in mice (Arnold et al. 2020).

### HMGB1 and CCl4-induced hepatotoxicity

A study has revealed that in liver fibrosis triggered by CCl4 in mice, HMGB1 expression is induced in the liver. Injection of an HMGB1 neutralizing antibody or recombinant HMGB1 may either decrease or promote liver fibrosis respectively. The HMGB1 neutralizing antibody effectively suppressed the synthesis of IL-6 and TNF-α triggered by CCl4 (Chen et al. 2014). Hepatocytes are the primary origin of HMGB1 in liver fibrosis induced by CCl4. Targeted elimination of HMGB1 specifically in hepatocytes partly prevented CCL4- induced hepatic fibrosis (Arriazu et al. 2017). These results indicate that HMGB1 is vital in hepatic fibrosis induced by CCl4. OPN may enhance this process (Arriazu et al. 2017). OPN from hepatocytes and HMGB1 specifically interact with hepatic stellate cells (HSCs), inducing their pro-fibrotic activity (Arriazu et al. 2017). The activation of NOX and inhibition of HDACs1/2 by OPN in HSCs can acetylate HMGB1 in vitro (Arriazu et al. 2017). HMGB1 interacts with TLR4 or RAGE, thus triggering the HSCs proliferation, migration and its profibrotic effects reacting to liver damage (Arriazu et al. 2017; Khanjarsim et al. 2017). HMGB1 activates pMEK1/2/pERK1/2/pcJun and PI3K/Akt axis, enhancing the synthesis of Collagen type I via RAGE (Arriazu et al. 2017; Ge et al. 2018). miRNAs are a class of small non-coding RNAs that can regulate post-transcriptional expression of genes. Among them, miR-146b can target Krüppel-like factor 4 (KLF4) to activate HSC, and hepatocyte nuclear factor 1A (HNF1A) to downregulate its expression, enhancing liver fibrosis. HMGB1 can upregulate the expression of miR-146b in a p65-dependent manner, thereby enhancing the impact of miR-146b (Ge et al. 2020). The generation of growth factor receptor-bound 2 (GRB2), which can be stimulated by HMGB1, enhances HSCs proliferation via the PI3K/AKT axis. (Ge et al. 2017). HMGB1 triggers endothelial to mesenchymal transition (EndoMT) and stimulates extracellular matrix (ECM) synthesis in human hepatic sinusoidal endothelial cells (HHSECs). Thus, HHSECs lose the capability of restraining HSCs activation (Wei et al. 2022). Collagen accumulation and EndoMT in liver as well as the levels of HMGB1 in serum were worsened in early growth response factor 1 (Egr1) knockout mice (Wei et al. 2022).

Astragali Radix (AR), curcumin, oxymatrine (OMT), ethyl pyruvate (EP), insulin-like growth factor 1 (IGF-1), chloroquine (CQ), as well as quercetin have may decrease CCl4-induced liver inflammation via suppressing HMGB1/TLR4/NF-κB axis(Dai et al. 2018; Li et al. 2016; Tu et al. 2012; Wen et al. 2021; Zhang et al. 2018b; Zhao et al. 2016; Zhao et al. 2021). The use of AR can greatly decrease the generation of type 1 collagen in mice via inhibiting HSCs activation and collagen secreted via HMGB1-RAGE-c-Jun pathway(Wen et al. 2021). γ-mangostin (γ-man) induces the activation of Sirtuin 3 (Sirt3), leading to an anti-fibrotic action. Sirt3 inhibits NAD(P)H oxidase (NOX) activity, which can activate histone deacetylase (HDAC1). As a consequence, the HMGB1 nucleus to cytoplasm transmission as well as autocrine stimulating action of HMGB1 is inhibited (Wang et al. 2019). However, γ-man inhibits the autocrine HMGB1-induced LX-2 cells activation (similar to HSCs in vivo) through the PI3K/AKT and MAPK p38 axis(Wang et al. 2019). In macrophages, 4-Octyl itaconate (OI) can promote the nuclear translocation of Nrf2and inhibit the nuclear translocation of NF-κB p65 induced by HMGB1, leading to the alleviation of ALF (Li et al. 2021). Nilotinib, a second-generation tyrosine kinase inhibitor, can improve liver fibrosis by reducing collagen accumulation. Nilotinib may enhance fibrotic via RAGE/HMGB1 axis and oxidative stress(Khanjarsim et al. 2017). Silymarin improves CCl4-induced liver fibrosis through enhancing of Egr1 accumulation in nucleus, decreasing serum HMGB1 levels, and inhibiting hepatic EndoMT in mice(Wei et al. 2022). Melatonin, (N-acetyl-5-methoxytryptamine, MLT), can reduce HMGB1 and IL-1α generation(Choi et al. 2015). In summary, some traditional Chinese medicines, herbal medicines, IGF-1, EP, OI, melatonin and tyrosine kinase inhibitors can down-regulate the serum levels of HMGB1, inhibit the activation of hepatic stellate cells, or inhibit the HMGB1 receptor and its related signaling pathways (such as RAGE and TLR4), thereby alleviating CCl4-induced liver injury.

### HMGB1 and drug-induced kidney injury

**Fig. 4:**
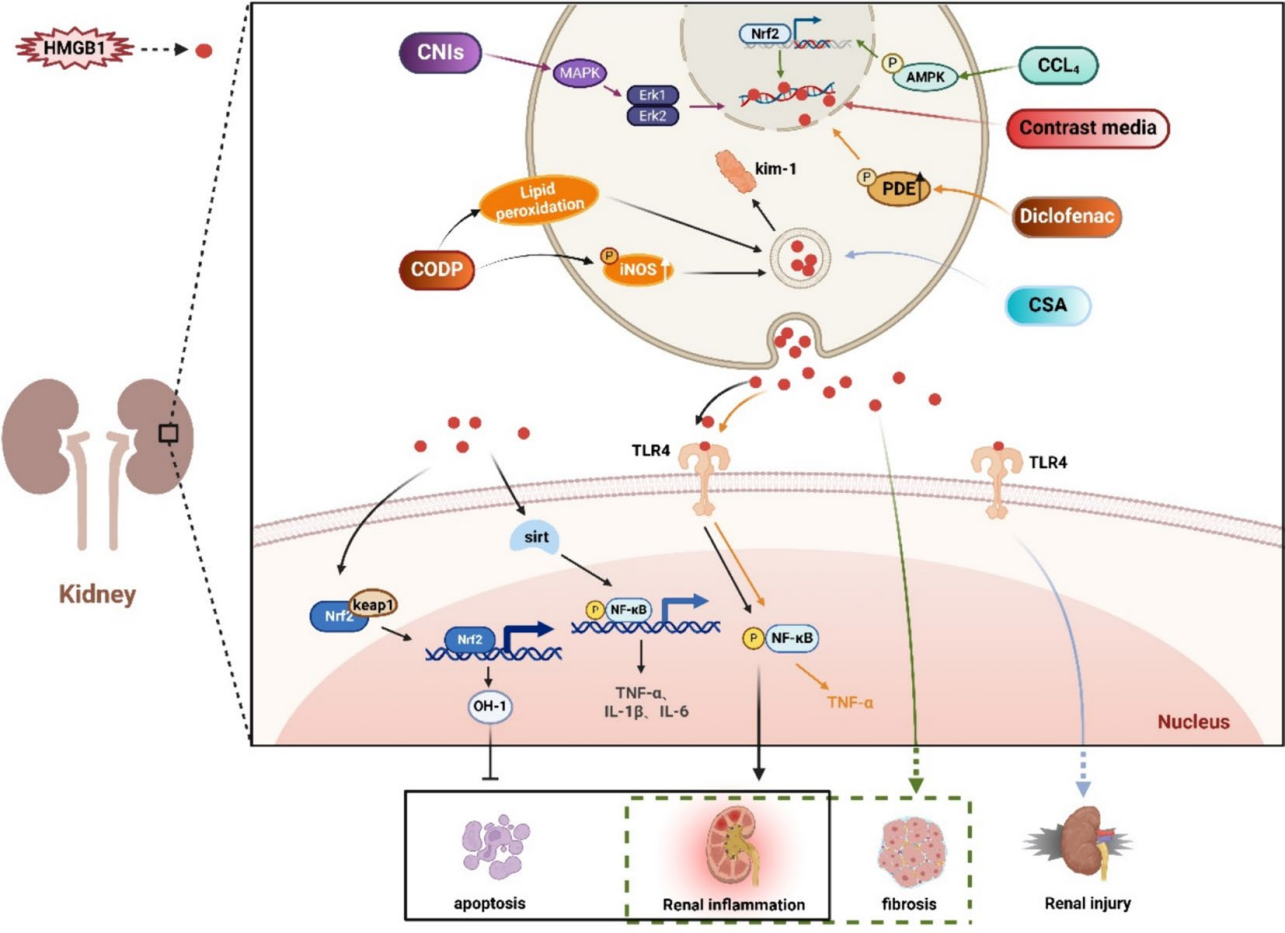
HMGB1 and Drug-Induced Kidney Injury. HMGB1 is involved in the process of kidney injury caused by drugs such as CDDP, diclofenac, contrast agents, CNIs, CsA and CCl4. 1) CDDP upregulates HMGB1 levels by promoting iNOS and lipid peroxidation. HMGB1 induced by CDDP causes tubular inflammation and apoptosis by upregulating the HMGB1/TLR4/NF-κB axis and downregulating the Keap1/Nrf2/HO-1 axis. At the same time, CDDP promotes the release of cytokines such as TNF-α, IL-6, and IL-1β by upregulating the Sirt1/NF-κB axis. The expression of Kim-1 was also upregulated. 2) Diclofenac and CsA-induced renal injury are related to HMGB1 and TLR4. HMGB1 is involved in contrast agent-induced acute kidney injury. CNIs upregulate HMGB1 levels through the MAPK/Erk1/2 pathway. AMPK/Nrf2/HMGB1 axis is involved in CCl4-induced renal fibrosis. This figure was drawn with Biorender (www.biorender.com)

Researches have demonstrated that in renal disorders, especially acute kidney injury, chronic kidney diseases, and primary glomerulonephritis, there is a significant presence of high HMGB1 expression in urine and renal tissues. This expression is found in various cell, such as kidney tubular epithelial cells, macrophages, as well as glomerular cells (Zhao et al. 2020). Pharmacotherapy-induced nephrotoxicity is a major contributor to the occurrence of acute kidney injury (AKI) in hospitals. Following sections provide a comprehensive explanation of the mechanisms by which HMGB1 facilitates drug-induced kidney damage (Fig. 4, HMGB1 and Drug-Induced Kidney Injury).

### HMGB1 and cisplatin-induced kidney injury

Cisplatin (CDDP) is a potent antineoplastic drug, however its significant nephrotoxicity restricts its clinical application. CDDP treatment results in increased iNOS and lipid peroxidation, while depleting the antioxidant response. Additionally, it elevates HMGB1/TLR4/NF-κB signaling pathway while downregulating Keap1/Nrf2/HO-1 axis, ultimately causing inflammation and apoptosis in kidney tubules (Michel and Menze 2019). Moreover, activation of the Sirt1/NF-κB axis induces the upregulation of pro-inflammatory cytokines such as TNF-α, NF-κB, IL-6 and IL-1β, in nephrotoxicity induced by CDDP(Yeung et al. 2004). Recent researches have demonstrated an increase in Kim-1 expression in renal tissues of rats treated with CDDP (Malik et al. 2015). Treatment with Tetramethylpyrazine (TMP) resulted in a considerable decrease in Kim-1 synthesis caused by CDDP. TMP and huaiqihuang (HQH) exert anti-inflammatory effects by stimulating PPAR-γ gene, leading to the suppression of the HMGB-1/TLR4/NF-κB axis, resulting in decreased iNOS and COX2, and dysregulating pro-inflammatory cytokines expression including TNF-α and IL-1β (Michel and Menze 2019; Oh et al. 2017). Activation of Nrf2/HO-1 axis by human growth hormone (hGH) may inhibit the HMGB-1/NF-κB axis in a IGF-1-dependent manner, potentially offering kidney protection during CDDP treatment (Mahran 2020). Ganoderma lucidum (GL), GA, and 18β GA may have similar impact (Mahran and Hassan 2020; Wu et al. 2015). Linalool has been demonstrated to reduce the production of inflammation- related factors and inhibit the HMBG1/TLR4 pathway in CDDP treatment (Mohamed et al. 2020). The protective effects of MSCs and blood mononuclear cells from human umbilical cord (hUCMSCs and hCBMNCs) were comparable in rats with CDDP-induced AKI. The observed protective effects may be linked to a decrease in HMGB1 level and a reduced Bax/Bcl-2 ratio (Xu et al. 2020), which is associated with the restriction of apoptosis and protection. However, how hUCMSCs and hCBMNCs cause downregulation of HMGB1 and reduction of Bax/Bcl-2 ratio requires further study. Whether there is a correlation between downregulation of HMGB1 and reduction of Bax/Bcl-2 ratio also needs further exploration. Several individual herbal remedies, including nelumbo nymphaea, may modulate HMGB1, NGAL, and Kim-1 responses to AKI- induced toxicity (Oh et al. 2017).

### HMGB1 and other drugs-induced kidney injury

Diclofenac- induced acute renal injury leads to a significant upregulation of mRNA expression of phosphodiesterase isoenzymes (1, 3, and 5), along with HMGB1, TLR4, NF-κB and TNF-α. phosphodiesterase inhibitors, such as pentoxifylline, vinpocetine, cilostazol, and sildenafil, may effectively reverse the alterations induced by diclofenac in renal tissues (Wadie et al. 2021). Contrast media notably increases both intracellular and serum HMGB1 levels. However, pretreatment with glycyrrhizin leads to a considerable drop in these levels. HMGB1 has a significant involvement in the progression of post-contrast acute kidney injury (PC-AKI). Glycyrrhizin, on the other hand, alleviates kidney malfunction via decreasing HMGB1 and oxidant stress (Oh et al. 2021). Calcineurin inhibitors (CNIs) are potent immunosuppressive drugs commonly used post-organ transplantation to prevent rejection. Persistent use of CNIs is associated with renal injury. CNIs induce a bioenergetic reprogramming by causing mitochondrial malfunction and promoting a transition to glycolysis. Subsequently, cell adhesion decreases, epithelial cell phenotype deteriorates, and HMGB1 is released. In vivo, CNIs stimulate tissular pro-remodeling signaling pathways. However, the administration of MAPK/Erk1/2 inhibitor has been shown to avert kidney damage, including decreasing HMGB1 secretion from renal epithelial cells and urine accumulation (Zmijewska et al. 2021). HMGB1 is essential in the progression of cyclosporine (CsA)-induced kidney damage via TLR4. Anti-HMGB1 antibody may enhance allograft survival in transplanted kidneys by protecting against chronic CsA-induced kidney injury (Park et al. 2016). Apart from its hepatic effects, CCl4 may also induce inflammation and fibrosis in the kidneys. Gastrodin alleviates kidney fibrosis and inflammation induced by CCl4 via the AMPK/Nrf2/HMGB1 axis (Ma et al. 2020a).

### HMGB1 and drug-induced myocardial injury

**Fig. 5:**
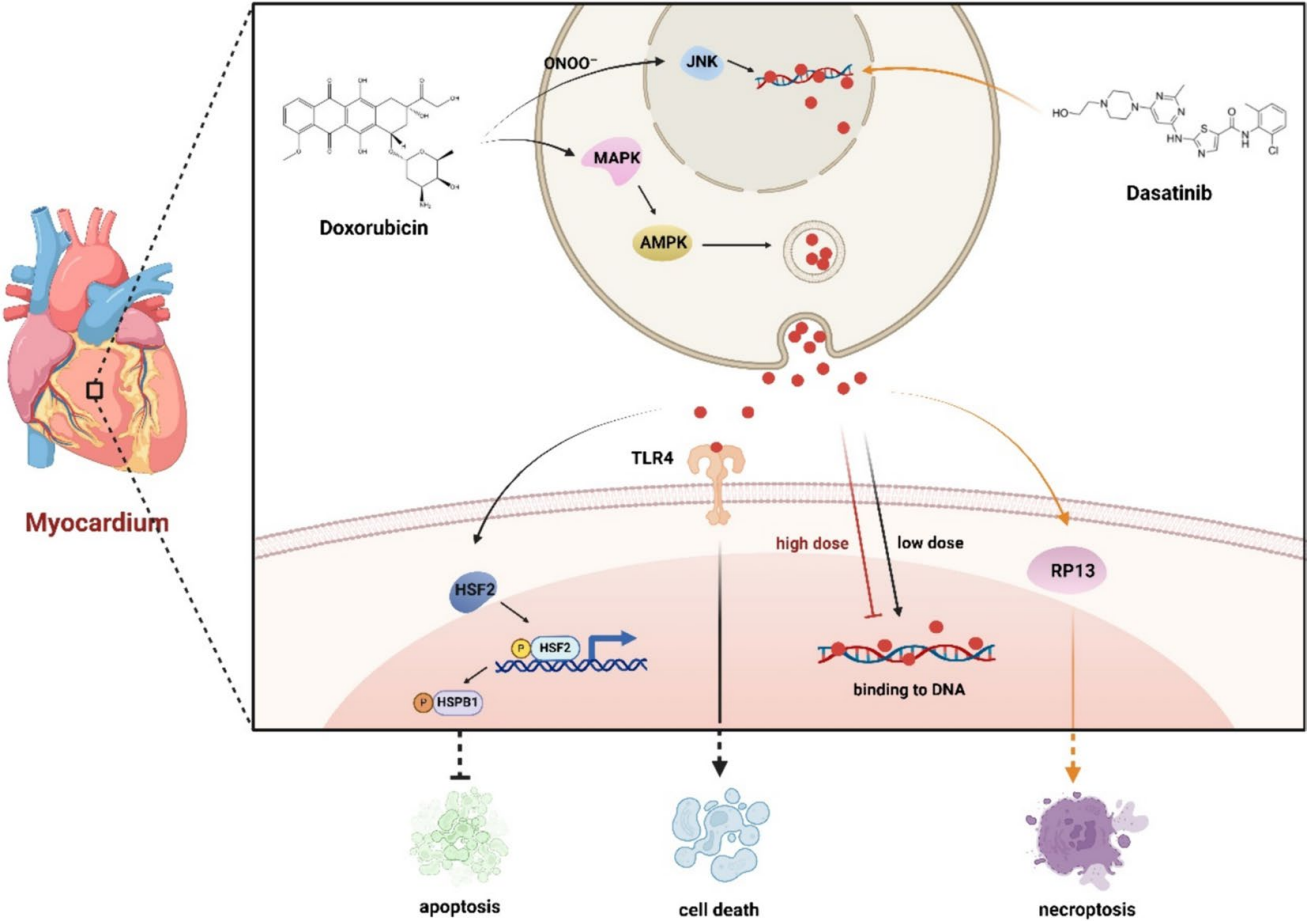
HMGB1 and Drug-Induced Myocardial Injury. HMGB1 is involved in cardiac damage caused by drugs such as DOX and Dasatinib. 1) DOX upregulates HMGB1 levels through the ONOO-/JNK pathway and MAPK/AMPK. HMGB1 induced by DOX induces apoptosis and autophagy through interaction with TLR4 and other pathways. Low doses of DOX can enhance the binding of HMGB1 and DNA, while high doses have the opposite effect. 2) RIP3 is involved in HMGB1-mediated renal injury caused by Dasatinib. This figure was drawn with Biorender (www.biorender.com)

The involvement of HMGB1 in cardiovascular diseases is controversial due to conflicting findings. Multiple studies have shown its association with damage to tissues in conditions including myocardial ischemia, heart failure, cardiac reperfusion injury, myocardial infarction, stress, diabetes, myocardial tissue inflammation, infection, and use of cardiotoxic chemotherapy drugs. However, other studies propose that HMGB1 also contributes to tissue repair and regeneration (Fig. 5, HMGB1 and Drug-Induced Myocardial Injury) (Pellegrini et al. 2019; Raucci et al. 2019). Activation of TLR9 is essential for wound healing, apoptosis, and angiogenesis after acute myocardial ischemia. TLR9 enhances the reparative fibrotic response of the heart by activating SMAD3. In TLR9-deficient mice, the expression of Bax, which promotes apoptosis, and the activation of caspase-3 are increased, while the expression of anti-apoptotic proteins Bcl-2 and Bcl-xl is decreased. In addition, TLR9 promotes HIF-1α expression through RelA, thereby enhancing VEGFA expression and angiogenesis. HMGB1 plays a crucial role in the above process by activating TLR9 (Liu et al. 2019). CpG-DNA or its analogs, synthetic oligonucleotides containing CpG motifs (CpG-ODN) promote TLR9 translocation from the endoplasmic reticulum to the endosome and thus be activated, while intracellular or extracellular HMGB1 can promote this process by binding to CpG-ODN (Ivanov et al. 2007). Cardiomyocyte injury and cardiac fibrosis are common adverse effects of novel medications, particularly anticancer medicines. Myocardial injury is often accompanied by elevated levels of aspartate transaminase (AST), lactate dehydrogenase (LDH), creatine kinase and its MB isoenzyme (CK-MB), cardiac troponins T (cTnT) (Antman 2018), ROS, and MDA (Ma et al. 2020b).

### HMGB1 and DOX-induced myocardial injury

DOX is an anti- tumor drug especially efficient for Hodgkin lymphoma, breast cancer, bladder cancer, and acute leukemia (Singal et al. 1992; Takemura and Fujiwara 2007). Nevertheless, DOX causes oxidative stress in cardiac cells, leading to lipid peroxidation and inhibiting nucleic acid and protein synthesis, eventually resulting in myocardial apoptosis (Ewer and Ewer 2010; Kalyanaraman et al. 2002; Zhu et al. 2009). Peroxynitrite (ONOO)-/c-Jun N terminal kinase (JNK) pathway is involved in DOX- induced cardiomyocyte apoptosis via HMGB1 (Yao et al. 2012). Researches have demonstrated the adverse impact of DOX on cardiac tissue and non-cancerous tissue. DOX affects the HMGB1/TLR4 axis via the MAPK/AMPK pathways, inducing apoptosis (Taskin et al. 2020). Moreover, inhibiting NF-κB through Met attenuated DOX- induced cardiomyocyte death (Alzokaky et al. 2023; Narumi et al. 2015). Another study has shown that HMGB1 is essential in Dox- induced cardiotoxicity by increasing autophagy, and its activity could be suppressed by the yes associated protein (YAP) (Luo et al. 2018b). In vivo, DOX has a bimodal effect on the intercalation of HMGB1 with DNA. At lower dosages, DOX enhanced the interaction between HMGB1 and DNA, while simultaneously reducing the interaction between the linker histone H1 and DNA. At elevated dosages, which align with the highest levels of DOX in the blood during chemotherapy, the binding of HMGB1 was also decreased (Bosire et al. 2022).

The long noncoding RNAs (lncRNA) colorectal neoplasia differentially expressed (CRNDE) derives from the hCG_1815491 locus on the 16th chromosome. It is situated on the opposite strand of neighboring iroquois homeobox transcription factor 5 (IRX5) gene. In an experiment, the levels of CRNDE decreased in a heart failure mouse model and HL-1 cells treated by DOX. CRNDE inhibited the cytoplasm translocation and acetylation, as well as the release of HMGB1 via deregulating PARP-1. Consequently, this restriction led to reduced apoptosis in myocardial cells and heart failure (Chen et al. 2020). miR-204 could alleviate cardiotoxicity triggered by DOX via blocking the HMGB1 pathway. Overexpression of miR-204 has a suppressive effect on apoptosis and autophagy triggered by DOX (Du et al. 2020). Rosuvastatin effectively reduced HMGB1 and RAGE production triggered by DOX in rat experiments (Zhang et al. 2018a). Glycyrrhizin (GL), a HMGB1 inhibitor, might mitigate cardiac injury triggered by DOX. GL could mitigate the growth-inhibiting effects of DOX in mice and reinstate the amount of AST, CK-MB, and superoxide dismutase (SOD). Furthermore, GL could enhance the impeded flow of autophagy in DOX-induced H9c2 cells by stimulating the disintegration of autolysosomes. The HMGB1-mediated Akt/mTOR pathway is a mechanism through which GL improves autophagy flow to avoid DIC (Lv et al. 2020).

### HMGB1 and other drugs-induced myocardial injury

Dasatinib has been frequently employed in imatinib- resistant chronic myelogenous leukemia and Ph^+^ acute lymphoblastic leukemia therapy(Force et al. 2007). Dasatinib- induced cardiotoxicity limit its wide clinical application(Cortes et al. 2016; Lamore et al. 2017; Will et al. 2008; Xu et al. 2018). RIP3-induced cardiomyocyte necroptosis has been proven to be the primary mechanism of dasatinib- induced cardiotoxicity. RIP3 knockdown partially reduces the necroptosis of cardiomyocytes induced by dasatinib. Comparable to the defensive properties of RIP3 knockdown, inhibiting HMGB1 also reduces necroptosis in cardiomyocytes induced by dasatinib, indicating that HMGB1 is essential in the cardiotoxicity of dasatinib via RIP3(Xu et al. 2018).

GA has the ability to inhibit cardiac fibrosis triggered by isoproterenol (ISO) via inhibiting the HMGB1/TLR2 signaling pathway, similar to its effect on liver fibrosis(Wu et al. 2018).

### HMGB1 and chemotherapy-induced peripheral neuropathy (CIPN)

**Fig. 6:**
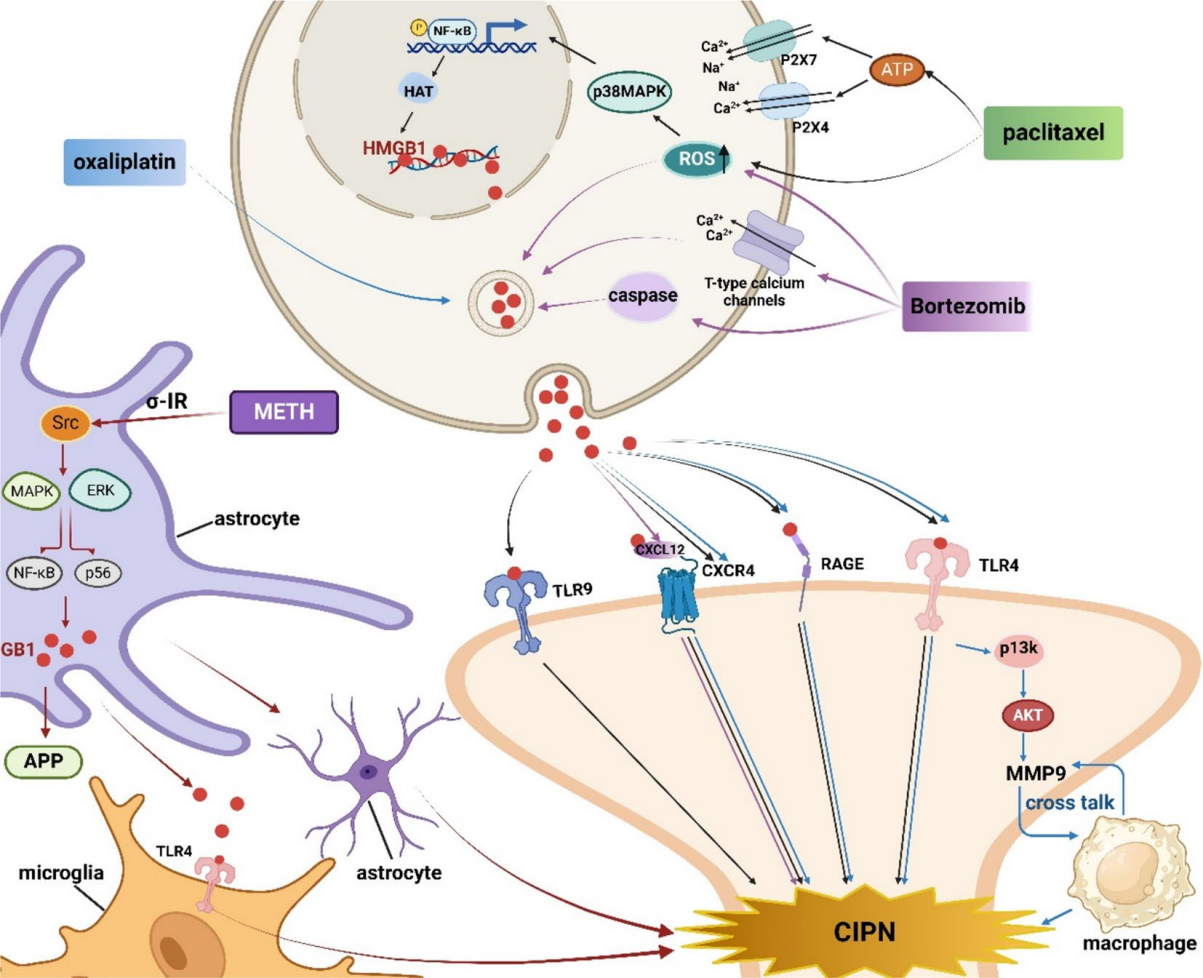
HMGB1 and Chemotherapy-Induced Peripheral Neuropathy. HMGB1 is involved in CIPN induced by drugs such as paclitaxel, oxaliplatin, bortezomib and METH. 1) Neuronal ATP stimulates P2X7 and P2X4 receptors during paclitaxel-induced CIPN, thereby triggering the release of HMGB1 from macrophages. Paclitaxel-induced HMGB1 inhibits CIPN by stimulating the ROS/p38 MAPK/NF-κB/HAT axis and interacting with receptors including RAGE, CXCR4, TLR4, and TLR9. 2) Oxaliplatin-induced HMGB1 promotes CIPN by interacting with receptors such as TLR4, RAGE and CXCR4, among which the HMGB-1-TLR4-PI3K/Akt-MMP-9 signaling pathway is crucial. 3) Bortezomib causes caspase-dependent release of HMGB1 from macrophages. Bortezomib-induced HMGB1 activates neurons by stimulating RAGE and stimulating the CXCL12/CXCR4 axis, leading to CIPN. 4) METH activates Src and MAPK/ERK axis through σ-1R receptor and further activates NF-κB and p65 to stimulate the production of HMGB1. HMGB1 induced by METH increases the expression of APP, activates astrocytes and microglia, and interacts with TLR4 to cause the deterioration of dopamine-producing nerve terminals in the striatum. This figure was drawn with Biorender (www.biorender.com)

CIPN, an adverse impact that limits the dosage of commonly used chemotherapy drugs including paclitaxel, oxaliplatin, vincristine, and bortezomib, worths attention, in which HMGB1 is of great significance in the emergence and progression (Fig. 6, HMGB1 and Chemotherapy-Induced Peripheral Neuropathy) (Nishida et al. 2016; Sekiguchi et al. 2018; Sekiguchi and Kawabata 2020; Tsubota et al. 2019; Tsujita et al. 2021). The use of a neutralizing antibody against HMGB1 (HMGB1-nAb) effectively inhibits the progression of CIPN in rodents. Additionally, the administration of thrombomodulin α (TMα, ART-123) promotes HMGB1 degradation and thus restraining the progress of CIPN in mice (Kotaka et al. 2020; Nishida et al. 2016; Tsubota et al. 2019; Tsubota et al. 2021).

### HMGB1 and paclitaxel-induced peripheral neuropathy

In paclitaxel- induced CIPN mice, macrophages, the primary cells secreting HMGB1, accumulate in the sciatic nerve and dorsal root ganglion (DRG) (Shibasaki et al. 2010). Liposomal clodronate depletion of resident macrophages alleviates CIPN in the sciatic nerve triggered by paclitaxel (Zhang et al. 2016). HMGB1 from macrophages contributes to paclitaxel- induced CIPN in mice via ROS/p38 MAPK/NF-κB/HAT axis (Sekiguchi et al. 2018). Extracellular HMGB1 is involved in the progression and maintenance of paclitaxel- triggered CIPN by activating RAGE, CXCR4, and TLR4 (Sekiguchi et al. 2018). miR-381, an antagonist of CXCR4, inhibits the progression of CIPN induced by paclitaxel (Zhan et al. 2018). TAK-242, a TLR4 antagonist, penetrates the central nervous system, effectively inhibits CIPN in C57BL/6 or Sprague–Dawley mice, but not in ddY mice. The effects of LPS and hyper-baric oxygen treatment (HBOT) are comparable (Wang et al. 2023). The use of N-acetylcysteine (NAC), an antioxidant, inhibits ROS formation and thereby decreases the occurrence of CIPN in patients receiving paclitaxel treatment (Khalefa et al. 2020). Neuronal ATP stimulates P2X7 and P2X4 receptors, triggering HMGB1 release from macrophages, which is involved in the progression of paclitaxel- induced CIPN mice. P2X7 and P2X4 antagonists suppress HMGB1 release from neuron-like cells and macrophages, thus prevents paclitaxel-induced CIPN in mice. Complete elimination of HMGB1 release from macrophages may be accomplished by depleting macrophages with liposomal clodronate or by treating them with minocycline and ethyl pyruvate, which inhibits HMGB1 release. Duloxetine, a P2X4 receptors inhibitor, has potential for treating existing CIPN and preventing CIPN in patients receiving paclitaxel (Domoto et al. 2022). TLR9, a receptor located inside cells, is essential in paclitaxel-induced CIPN only in male mice, indicating a sex dimorphism of TLR9. Similarly, TLR9 induces TNF and CXCL1 secretion in paclitaxel-induced CIPN from macrophages only in male mice (Luo et al. 2019). TLR4-mediated pain perception has a similar phenomenon (Luo et al. 2019).

### HMGB1 and oxaliplatin-induced peripheral neuropathy

Oxaliplatin, a commonly administered platinum-based chemotherapeutic drug for treating various malignancies such as colorectal and gastrointestinal cancers (André et al. 2004; Cassidy et al. 2008; de Gramont et al. 2000; Schmoll et al. 2015), induces acute CIPN in nearly all patients, characterized by the development of cold allodynia (Grothey 2003). This acute condition often progresses to chronic CIPN, characterized by sensations of tingling and numbness (Grothey 2005). In non-macrophage cells, oxaliplatin-induced CIPN may be triggered by HMGB1 through TLR4, RAGE, and CXCR4. TMα efficiently mitigates oxaliplatin- induced CIPN in a thrombin-dependent manner. HMGB1-nAb or TMα may be provided to neutralize HMGB1 and prevent CIPN in mice undergoing oxaliplatin treatment. Furthermore, blocking RAGE, CXCR4, or TLR4 using pharmacological methods can also prevent CIPN in these rodents (Kotaka et al. 2020; Tsubota et al. 2019). The HMGB-1-TLR4-PI3K/Akt-MMP-9 signaling pathway appears to have a significant impact on the interaction between macrophages and neurons in CIPN mice. MMP-9 holds promise as a target for developing therapeutic interventions for CIPN. NAC decreased the production of calcitonin gene-related peptide (CGRP), a pain marker, in the spinal cord by inhibiting MMP-9/2 activities (Gu et al. 2020).

### HMGB1 and bortezomib-induced peripheral neuropathy

Chemotherapy drugs for multiple myeloma, such as bortezomib, a proteasome inhibitor, frequently induces CIPN. The production of ROS and T-type calcium channels, especially the Cav3.2 variant, has been shown to be implicated in bortezomib-induced CIPN. Multiple doses of bortezomib lead to release of HMGB1 from macrophages in a caspase-dependent manner, which then activates neurons via stimulating RAGE and expediting CXCL12/CXCR4 axis, contributing to CIPN. However, this mechanism does not involve the activation of TLR4 or TLR5. In macrophages, bortezomib stimulates caspase activation and apoptosis, resulting in caspase-dependent HMGB1 release. In bortezomib-induced CIPN, repeated administration of a caspase inhibitor effectively reduces the CIPN caused by bortezomib. This effect can be reversed by a neutralizing antibody or TMα, which deactivates HMGB1 (Tsubota et al. 2021).

### HMGB1 and methamphetamine-induced neuroinflammatory

Methamphetamine (METH), a frequently abused drug, leads to the deterioration of nerve terminals in the striatum, which generate dopamine. HMGB1 play a vital role in the neurotoxic effects induced by METH. A study in vivo demonstrated that the intricate molecular mechanism of METH- induced astrocyte activation and migration occur via σ-1R receptor, activating Src and MAPK/ERK axis. Consequently, NF-κB p65 is activated, thereby stimulating astrocytes to express and actively secrete HMGB1 (Zhang et al. 2015c). After exposure to METH, neurons generate HMGB1, which might potentially activate neighboring astrocytes and microglia, leading to the promotion of neuroinflammation. METH may induce a microglial inflammatory response by stimulating the TLR4 signaling pathway (Frank et al. 2016). The expression of amyloid-beta precursor protein (APP) may also be increased by METH-induced HMGB1 expression. Inhibiting HMGB1 in the pathway resulted in an inhibition of APP expression (Alabed et al. 2021). Administration of anti-HMGB1 mAb intravenously leads to the reduction of METH-induced hyperthermia, activation of microglia, release of HMGB1 from neuronal nuclei in central nervous system, and neurotoxicity in striatum (Masai et al. 2021).

### HMGB1 and drug-induced lung toxicity

**Fig. 7:**
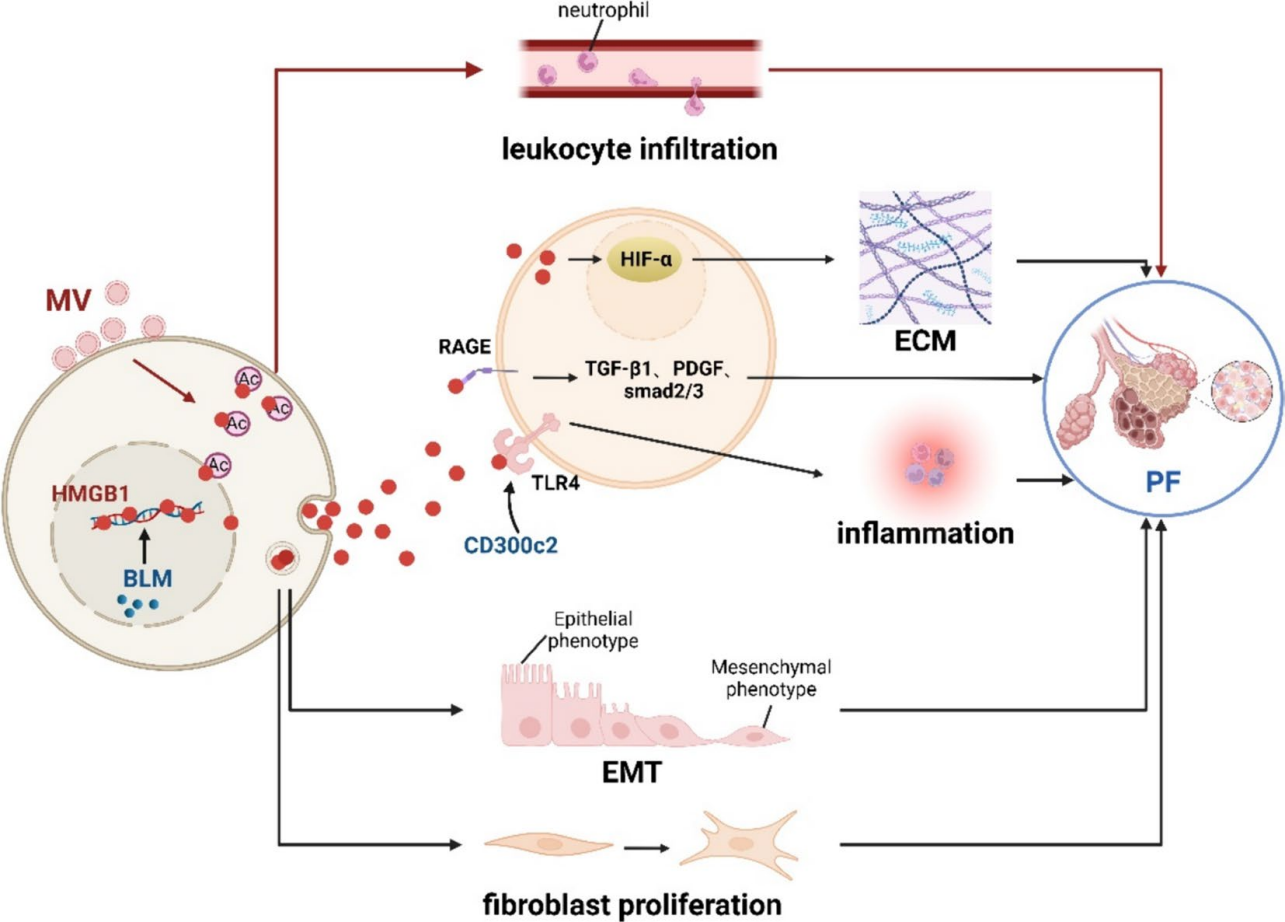
HMGB1 and Drug-Induced Lung Toxicity. Pulmonary fibrosis is the most noteworthy side effect of BLM, and HMGB1 plays an important role in this process. 1) BLM-induced HMGB1 induces fibroblast proliferation through RAGE and increases pro-fibrotic cytokines such as TGF-β1 and PDGF in the lungs. 2) TLR4 is also involved in HMGB1-mediated BLM-induced pulmonary fibrosis, and CD300c2 enhances this process. 3) HMGB1 stimulated by BLM induces high expression of HIF1-α, resulting in enhanced aerobic glycolysis and extracellular matrix formation. 4) The TGF-β1/Smad2/3 axis is involved in HMGB1-triggered EMT in BLM-induced pulmonary fibrosis. This figure was drawn with Biorender (www.biorender.com)

In this section, we detail the HMGB1- mediated pulmonary toxicity of drugs (Fig. 7, HMGB1 and Drug- Induced Lung Toxicity).

### HMGB1 and bleomycin (BLM)-induced lung toxicity

BLM, an antineoplastic agent used to treat various types of tumors, including reproductive system tumors and malignant pleural effusions (Della Latta et al. 2015). Lung injury, including pulmonary fibrosis (PF), is the most noteworthy side effect of BLM (Della Latta et al. 2015). In idiopathic pulmonary fibrosis (IPF) and hypersensitivity pneumonitis (HP), the HMGB1 levels in bronchoalveolar lavage fluid (BALF) are increased compared to the control group. HMGB1 has the ability to directly induce fibroblast proliferation in vitro (Hamada et al. 2008). In alveolar type II epithelial cells of mice, BLM can induce epithelial-to-mesenchymal transition (EMT) in co-culture with HMGB1. Although RAGE^−/−^ mice had a similar initial inflammatory reaction, they were mostly protected from the subsequent fibrotic effects induced by BLM. These findings suggest that HMGB1, which is likely generated by inflammatory cells, activates RAGE signaling in response to BLM-induced effects. This signaling increases profibrotic cytokines in the lungs, such as TGF-β1 and PDGF (He et al. 2007). Administration of anti-HMGB1 antibody or ethyl pyruvate inhibits inflammation, apoptosis, and fibrosis, thus alleviating BLM-induced lung fibrosis (Hamada et al. 2008). Nevertheless, administering of soluble RAGE did not ameliorate fibrosis (Englert et al. 2011).

A RAGE-antagonist peptide (RAP) has been recently synthesized, targeting the binding site of HMGB1 to RAGE. In a mouse model of IPF triggered by BLM, RAP effectively deregulates molecules involved in fibrosis including hydroxyproline, TGF-β, α-SMA, suggesting that RAP has the potential to treat IPF (Piao et al. 2022). CD300c2, (MAIR-II, LMIR2 or CLM-4), an immunoglobulin-like receptor existing in cell membrane of immune cells such as lymphocytes, monocytes and macrophages, enhances the inflammatory reactions triggered by HMGB1 and TLR4 in lung damage produced by BLM. CD300c2 also increased the generation of a substance that attracts neutrophils from macrophages, which was produced by HMGB-1 stimulation following BLM administration (Nakazawa et al. 2019). Another study demonstrated that HMGB1 induces high HIF1-α expression, resulting in an elevation of aerobic glycolysis and enhanced formation of extracellular matrix in PF (Xu et al. 2017). Studies have demonstrated that TGF-β1/Smad2/3 axis is involved in EMT triggered by HMGB1 in BLM-induced pulmonary fibrosis (Li et al. 2015).

GA, a HMGB1 inhibitor, has been demonstrated to alleviate pulmonary injury triggered by BLM via MAPK and Smad3 in mice (Zhu et al. 2021). Gefitinib has the potential to alleviate lung fibrosis induced by BLM via the HMGB1/NOXs-ROS/EGFR-MAPKs-AP-1/NF-κB pathway (Li et al. 2018). Itraconazole may counteract lung fibrosis induced by BLM through decreasing oxidative stress, modulating the HMGB1, TLR4 NLRP3 and NF-κB signaling, and affecting the balance between autophagy and apoptosis (Elkhoely et al. 2023). Protocatechuic aldehyde reduces pulmonary fibrosis via inhibiting the HMGB1/RAGE pathway (Zhang et al. 2015a). Atazanavir sulfate reverses cell proliferation induced by EMT and prevents pulmonary fibrosis by inhibiting HMGB1/TLR signaling (Song et al. 2018). Other drugs that inhibit HMGB1 levels and hence inhibit BLM-induced lung fibrosis include thrombomodulin, fatty acid, nitrogenes, astragaloside IV, Yupingfeng, pulmonary rehabilitation mixture, dioscin, simvastatin, and some fragments of the depolymerized heparins (Cui et al. 2015; Kida et al. 2018; Li et al. 2017; Liu et al. 2014; Wilkinson et al. 2020; Wu and Wang 2019; Yan et al. 2017; Zhang et al. 2015b). Nevertheless, further research is required.

### HMGB1 and drug-induced gastrointestinal toxicity

**Fig. 8:**
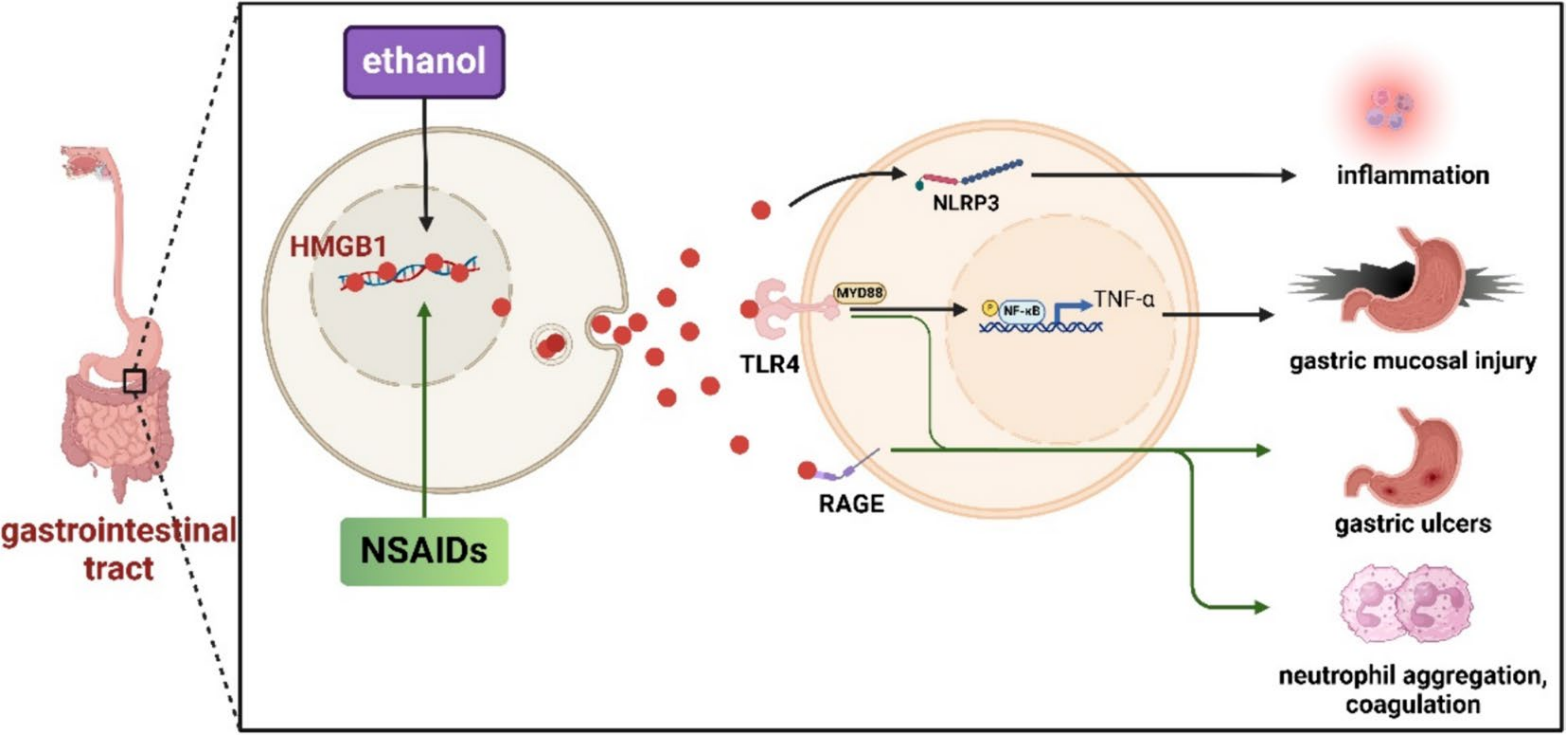
HMGB1 and Drug-induced Gastrointestinal Toxicity. HMGB1 plays an important role in the gastrointestinal damage caused by ethanol and NSAIDs. 1) HMGB1 produced by ethanol stimulation can upregulate NF-kB and TNF-α by stimulating MyD88/TLR4. Ethanol-stimulated production of HMGB1 can also stimulate the production of NLRP3. 2) NSAIDs can cause gastric ulcers, bleeding and perforation by interacting with TLR4 and RAGE. This figure was drawn with Biorender (www.biorender.com)

HMGB1 elicits gastrointestinal toxicity through various signaling pathways. Currently, multiple substances exhibiting gastrointestinal toxicity have been substantiated to have mechanisms associated with HMGB1, such as non-steroidal anti-inflammatory drugs (NSAID) and ethanol, etc. (Fig. 8, HMGB1 and Drug-induced Gastrointestinal Toxicity).

### HMGB1 and ethylalcohol-induced gastrointestinal toxicity

Researchers have shown a significant association between HMGB1 and the gastrointestinal toxicity induced by ethanol. Following ethanol administerion, HMGB1 expression in the tissues markedly increases, while Nrf2 expression decreases significantly. This is because HMGB1 can stimulate NF-kB and TNF-α up-regulation via TLR4 receptors. However, this process can be reversed by Raspberry ketone (RK) (Badr et al. 2019). Additionally, activation of TLRs signaling pathways can promote autophagy by activating p62. Keap1 degradation is enhanced during autophagy, thereby activating the Nrf2 pathway (Yin and Cao 2015). Atypical expression of TLR4 is essential in the gastrointestinal toxicity induced by HMGB1. The findings revealed that both downregulation and upregulation of TLR4 expression contributed to ethylalcohol-induced gastric mucosal damage. The TLR4/MyD88/NF-κB axis is essential in ethanol-triggered gastric mucosal injury pathogenesis (Ye et al. 2013). Myeloid differentiation Factor 88 (MyD88) is involved in regulating TLR4 signaling. MyD88-mediated signaling leads to κB inhibitor (IkB) phosphorylation, hampering its inhibitory effects in NF-kB. Upon exposure to an external signal, cells release NF-κB from the inhibitory complex, allowing it to promptly enter the nucleus and initiate the transcription of genes that encode inflammatory cytokines (Hayden and Ghosh 2008; Wang et al. 2011). Other studies demonstrated that animals lacking the TLR4 gene had gastrointestinal injury that was no longer influenced by exogenous HMGB1 (Nadatani et al. 2012). The findings demonstrated that the introduction of exogenous HMGB1 increased small intestine damage in Rag-KO and TLR2-KO mice, and increased TNF-α mRNA expression in TLR2-KO mice. However, there was no effect on TLR4-KO mice injury and TNF-α mRNA expression, suggesting that HMGB1 induces small intestine damage by activating TLR4-dependent signaling pathways. Additionally, HMGB1 is essential in the gastrointestinal harm induced by ethanol via stimulating HMGB1, NLRP3, and NF-κB (Alzokaky et al. 2020). Furthermore, NLRP3 is associated with inflammation, including diabetes, arteriosclerosis and inflammatory bowel disease (Duewell et al. 2010; Lee et al. 2013; Villani et al. 2009). C-phycocyanin inhibits this process and improves the prognosis (Alzokaky et al. 2020).

### HMGB1 and NSAID-induced gastrointestinal toxicity

NSAIDs are frequently used in clinical practice to alleviate pain and inflammation associated with rheumatic diseases and osteoarthritis. However, the administration of NSAIDs may often lead to the development of gastric ulcers, bleeding, and perforations (Adachi et al. 2011; Mahmoud and Abd El-Ghffar 2019). HMGB1 has been shown to induce NASID-mediated gastrointestinal toxicity via TLR4 (Nadatani et al. 2012; Nadatani et al. 2013). HMGB1 is a complex factor in gastric ulcer healing. Exogenous HMGB1 delays gastric ulcer healing, while immune neutralization of HMGB1 or inhibition of HMGB1 release promotes ulcer healing. The delay of gastric ulcer by HMGB1 is RAGE- and TLR4-dependent. HMGB1 accelerates the wound healing process and regeneration by enhancing the migration of skin fibroblasts and keratinocytes in a RAGE-dependent manner. TNFα overexpression and excessive neutrophil infiltration are important factors in delayed healing of gastric ulcers, while exogenous HMGB1 induces TNFα expression and Myeloperoxidase (MPO) activity (Nadatani et al. 2013). Histopathological observation proved that HMGB1 could mediate local neutrophil aggregation and coagulation activation through the above inflammatory factors (van Zoelen et al. 2009).

### Therapeutical strategies for drug-induced adverse reactions

Summary of the protective agents against organic toxicities by targeting HMGB1 is in Table 3 (Therapeutical Strategies for Drug-Induced Adverse Reactions Mediated by HMGB1).

**Table 3:** Therapeutical Strategies for Drug-Induced Adverse Reactions Mediated by HMGB1

Organ	Representative drug	Promising candidate	Regulatory mechanisms	REF
Liver	Ethylalcohol	HMGB1 ablation or knockdown	-	(Gaskell et al. 2018; Khambu et al. 2019)
		Digitoflavone, Betaine, SIRT1	SREBP1, PPARα and TLR4 inhibition	(Lan et al. 2020; Shang et al. 2022)
		Salvianic acid A	up‐regulation of BRD4	(Zhao et al. 2022)
	APAP	Glycyrrhizin	HMGB1-TLR4-IL-23-IL-17A axis inhibition	(Yang et al. 2014)
		Neutrophil depletion or NETs	HMGB1 release inhibition	(Liu et al. 2021a)
		h2G7	HMGB1 neutralizing antibody	(Lundbäck et al. 2016)
		HMGB1 neutralization	NF-κB activation	(Yang et al. 2012)
		Metformin	Interacting with acidic tail of HMGB1	(Horiuchi et al. 2017)
		Glycyrrhetinic acid	TLR4-IRAK1-MAPK/NFκB inhibition	(Yang et al. 2017)
		Benzyl alcohol, diacerein, capsaicin, kaempferol	TLR4 inhibition, JNK phosphorylation inhibition, inflammasome activity inhibition	(Cai et al. 2014)
		Diacerein, DAPT	Increased expression of PPAR-γ	(Cai et al. 2014)
		Apoptosis repressor with ARC	Release inhibition of HMGB1	(An et al. 2013)
		Liuweiwuling tablets	Decreased HMGB1, TNF-α and IL-1β levels	(Fink 1990)
		Berberine	Inhibiting HMGB1, phosphorylated NF-κB and other inflammatory cytokines expression	(Zhao et al. 2018)
		Chikusetsusaponin V	neutrophil NETs, HMGB1 and Caspase-1 inhibition	(Liu et al. 2021a)
		18-mer-HP	RAGE inhibition	(Arnold et al. 2020)
	CCl4	HMGB1 neutralizing antibody, recombinant HMGB1	TNF-α and IL-6 generation inhibition	(Chen et al. 2014)
		Ablation of HMGB1 in hepatocytes	-	(Arriazu et al. 2017)
		Astragali Radix, curcumin, oxymatrine, ethyl pyruvate, insulin-like growth factor 1, chloroquine, quercetin	TLR4/NF-κB inhibition	(Li et al. 2016; Tu et al. 2012; Wen et al. 2021; Zhang et al. 2018b; Zhao et al. 2016; Zhao et al. 2021)
		Astragali Radix	HMGB1-RAGE-c-Jun inhibition to decrease HSCs activation and collagen generation	(Wen et al. 2021)
		γ-man	Activating SIRT3 to inhibit HMGB1 nucleus to cytoplasm translocation and inhibiting the self-stimulating effect of HMGB1 by inhibiting NAD(P)H oxidase (NOX) activity, which in turn activates histone deacetylase (HDAC1), inhibiting HSCs via the PI3K/AKT and MAPK p38 axis	(Wang et al. 2019)
		4-Octyl itaconate	Enhancement of Nrf2 nuclear translocation, NF-κB p65 nuclear translocation inhibition	(Li et al. 2021)
		Nilotinib	RAGE and oxidative stress inhibition	(Khanjarsim et al. 2017)
		Silymarin	Egr1 nuclear accumulation, hepatic EndoMT inhibition, decreasing serum HMGB1 content	(Wei et al. 2022)
		Melatonin	HMGB1 and IL-1α release inhibition	(Choi et al. 2015)
Kidney	CDDP	TMP, huaiqihuang	Increasing PPAR-γ gene expression to suppress TLR4/NF-κB axis	(Michel and Menze 2019; Oh et al. 2017)
		hGH, Ganoderma lucidum, glycyrrhetinic acid, 18βGA	Inhibit the HMGB-1/NF-κB axis IGF-1dependently through activating Nrf2/HO-1	(Mahran 2020; Mahran and Hassan 2020; Wu et al. 2015)
		Linalool	Diminishing TLR4, MYD88, and TRIF expressions	(Mohamed et al. 2020)
		hUCMSCs, hCBMNCs	HMGB1 downregulation, decreasing Bax/Bcl-2 ratio	(Xu et al. 2020)
		Single herbal medicines (nelumbo nymphaea)	-	(Oh et al. 2017)
	Diclofenac	phosphodiesterase inhibitors (pentoxifylline, vinpocetine, cilostazol and sildenafil)	HMGB1, TLR4, NF-κB and TNF-α inhibition	(Wadie et al. 2021)
	Iodinated contrast media	Glycyrrhizin	HMGB1 inhibition	(Oh et al. 2021)
	Calcineurin inhibitors	MAPK/Erk1/2 inhibitor	HMGB1 inhibition	(Zmijewska et al. 2021)
	Cyclosporine	Anti-HMGB1 antibody	-	(Park et al. 2016)
	CCl4	Gastrodin	AMPK/Nrf2/HMGB1	(Ma et al. 2020a)
Myocardium	Doxorubicin	Met	NFκB inhibition	(Alzokaky et al. 2023)
		Yes associated protein, miR-204	Autophagy inhibition	(Du et al. 2020; Luo et al. 2018b)
		CRNDE	PARP-1 inhibition to inhibit HMGB1 acetylation	(Chen et al. 2020)
		Rosuvastatin	HMGB1 and RAGE inhibition	(Zhang et al. 2018a)
		Glycyrrhizin	Accelerating autolysosome degradation	(Lv et al. 2020)
	Dasatinib	RIP3 knockdown, inhibition of HMGB1	-	(Xu et al. 2018)
	Isoproterenol	Glycyrrhetinic acid	TLR2 inhibition	(Wu et al. 2018)
Peripheral nerve	Paclitaxel, Oxaliplatin, Bortezomib	Oluble thrombomodulin (TMα, ART-123、recomodulin®)	Thrombin-dependent HMGB1 degradation	(Kotaka et al. 2020; Nishida et al. 2016; Tsubota et al. 2019; Tsubota et al. 2021)
	Paclitaxel	Liposomal clodronate	Macrophage accumulation inhibition	(Zhang et al. 2016)
		miR-381	CXCR4 antagonists	(Zhan et al. 2018)
		TAK-242, hyper-baric oxygen therapy	TLR4 antagonist	(Wang et al. 2023)
		P2X7 antagonists, P2X4 antagonists (Duloxetine)	HMGB1 release inhibition	(Domoto et al. 2022)
	Oxaliplatin	NAC	MMP-9/2 inhibition	(Gu et al. 2020)
	Bortezomib	Caspase inhibitor	-	(Tsubota et al. 2021)
	Methamphetamine	Blocking HMGB1	APP expression inhibition	(Alabed et al. 2021)
		anti-HMGB1 mAb	-	(Masai et al. 2021)
Lung	Bleomycin	Anti-HMGB1 antibody, ethyl pyruvate	Attenuating inflammation, apoptosis and fibrosis	(Hamada et al. 2008)
		RAGE-antagonist peptide	RAGE inhibition	(Piao et al. 2022)
		Glycyrrhizic acid	MAPK and Smad3 pathways	(Zhu et al. 2021)
		Gefitinib	HMGB1/NOXs-ROS/EGFR-MAPKs-AP-1/NF-κB pathways inhibition	(Li et al. 2018)
		Itraconazole	Alleviate oxidative stress, regulating HMGB1/TLR4 axis and NLRP3and NF-κB	(Elkhoely et al. 2023)
		Protocatechuic aldehyde	HMGB1/RAGE	(Zhang et al. 2015a)
		Atazanavir sulphate	HMGB1/TLR inhibition	(Song et al. 2018)
		Thrombomodulin, fatty acid nitrogenes, astragaloside IV, Yupingfeng, pulmonary rehabilitation mixture, dioscin, simvastatin and some fragments of the depolymerized heparins	Need further exploration	(Cui et al. 2015; Kida et al. 2018; Li et al. 2017; Liu et al. 2014; Wilkinson et al. 2020; Wu and Wang 2019; Yan et al. 2017; Zhang et al. 2015b)
	Hyperoxia	EP, GTS-21, ascorbic acid, exogenous surfactant, N-acetylcystein	HMGB1 inhibition	(Bezerra et al. 2019; Patel et al. 2020; Qiao et al. 2019; Sitapara et al. 2020a; Sitapara et al. 2021; Sitapara et al. 2020b)
		Enoxaparin	Akt pathway inhibition	(Li et al. 2011)
Gastrointestinal tract	Ethanol	Raspberry ketone	TLR4-NFκB-TNFα inhibition	(Badr et al. 2019)
		C-phycocyanin	HMGB1 / NLRP3 / NF-κB pathway inhibition	(Alzokaky et al. 2020)

### Perspectives and conclusion

HMGB1 is undoubted a crucial molecule that plays a vital role in numerous drug-induced toxic reactions, such as those caused by APAP, cisplatin, doxorubicin, oxaliplatin, paclitaxel, and bleomycin. Additionally, the receptors of HMGB1, namely RAGE, TLRs, and CXCR4, have also been established as pivotal factors in the drug-induced toxicity mediated by HMGB1. Excessive or uncontrolled release of HMGB1 and activation of HMGB1 receptors can lead to increased inflammatory responses and cell death, including increased cytokine production, inflammasome activation, pyroptosis, NETs formation, and migration of leukocytes and fibroblasts. These effects manifest in organs as inflammation, fibrosis and sclerosis, tissue degeneration, necrosis, functional impairment, pain, numbness, gastrointestinal ulceration or perforation, and gastrointestinal bleeding.

However, there are numerous inquiries that warrant further investigation. For instance, it remains to be determined how post-translational modifications of HMGB1 affects its functions. HMGB1 manifests in three distinct redox forms. It is yet to be elucidated whether the mode of extracellular secretion of HMGB1 (active or passive) impacts its redox form. Additionally, it remains to be explored whether the different forms of HMGB1 affect its binding to the receptor and subsequent extracellular functions. Lastly, it is imperative to ascertain whether the redox form of HMGB1 influences the toxicity of drugs induced by HMGB1.

Given that RAGE, TLRs, and CXCR4 trigger drug-induced organic injury induced by HMGB1, it is important to investigate their involvement in specific instances of organic injury. For instance, HMGB1-TLR4-IL-23-IL-17A axis is identified as essential in APAP-induced liver injury. Thus, it is pertinent to determine whether RAGE and CXCR4 are also involved in APAP-induced liver damage, and whether HMGB1-TLR4 axis participates in organic damage induced by other drugs, such as myocardial injury. Additionally, further exploration is needed to ascertain which receptor plays the most vital role in drug-induced organic damage, necessitating additional research.

Furthermore, it is worth noting that most proposed therapeutic approaches targeting HMGB1 primarily focus on HMGB1 antagonism and receptor gene knockout. Further development is required for drugs that antagonize HMGB1. Additionally, the mechanisms underlying the action of numerous drugs targeting HMGB1 necessitate further exploration. Furthermore, the applicability of these drugs in clinical practice requires evaluation through additional clinical trials. It is worth noting that HMGB1 has the potential to enhance the effectiveness of certain drugs, as evidenced by its ability to augment the cytotoxic impact of cisplatin on tumor cells. The identification of drugs that can enhance HMGB1's efficacy also warrants further investigation. Lastly, investigating the role of other members of the HMGB protein family, such as HMGB2 and HMGB3, can serve as a promising avenue for future research.

## Data Availability

No datasets were generated or analyzed during the current study.
